# Development of Pharmabiotic Gel-Serums with *Lacticaseibacillus casei* Postbiotics and Paraprobiotics Using Chitosan and Carbopol

**DOI:** 10.3390/gels12090838

**Published:** 2026-09-13

**Authors:** Pervin Soyer, A. Alper Öztürk

**Affiliations:** 1 Department of Pharmaceutical Microbiology, Faculty of Pharmacy, Anadolu University, Eskisehir 26470, Turkey; pervinsoyer@anadolu.edu.tr; 2 Department of Pharmaceutical Technology, Faculty of Pharmacy, Anadolu University, Eskisehir 26470, Turkey

**Keywords:** *Lacticaseibacillus casei*, postbiotic, paraprobiotic, pharmabiotic gel-serum, chitosan, Carbopol^®^, antimicrobial activity, antibiofilm activity, antioxidant activity

## Abstract

Postbiotic and paraprobiotic preparations are increasingly investigated as non-viable microbial-derived components for topical formulation development, offering an alternative to systems containing live microorganisms. However, their physicochemical and biological performance may depend strongly on the polymeric carrier, and direct comparisons of postbiotic and paraprobiotic fractions derived from the same microbial source in different gel matrices remain limited. This study aimed to develop and comparatively evaluate Carbopol^®^- and chitosan-based pharmabiotic gel-serums containing *Lacticaseibacillus casei*-derived postbiotic (PB) and paraprobiotic (PPB) fractions. The formulations were characterized in terms of macroscopic appearance, pH, spreadability, and rheological behavior, and their antimicrobial activity, effects on preformed biofilm biomass, and DPPH radical-scavenging activity were evaluated. Blank Carbopol^®^ and chitosan formulations and free PB and PPB fractions were included as controls to distinguish formulation-matrix-associated effects from those observed for the complete formulations. The developed gel-serums exhibited homogeneous initial macroscopic characteristics, pH values ranging from 4.22 to 5.66, and pseudoplastic shear-thinning behavior. Among the PB-containing systems, CA-CS-PB exhibited pronounced antimicrobial activity, with inhibition-zone diameters of 18.00 mm against *Candida albicans* and 19.93 mm against *Candida krusei*, and MIC values of 1156 μg/mL against both species. CA-CS-PB also reduced preformed *Staphylococcus aureus* and *Pseudomonas aeruginosa* biofilm biomass by 92.89% and 87.63%, respectively, and exhibited the highest DPPH radical-scavenging activity (73.50%). Among the PPB-containing systems, CA-C-PPB produced reductions of 94.63% and 93.71% in preformed *S. aureus* and *P. aeruginosa* biofilm biomass, respectively. Blank formulations also exhibited measurable biological responses, indicating that the activities of the complete formulations should be interpreted as formulation-level effects rather than being attributed exclusively to the incorporated PB or PPB fractions. Because PB and PPB were incorporated at different concentrations (5% and 1% *w*/*w*, respectively), these findings should not be interpreted as an equivalent-dose comparison of their intrinsic biological potency. Overall, the findings demonstrate the feasibility of incorporating *L. casei*-derived PB and PPB fractions into different polymeric gel-serum systems and highlight the influence of both the pharmabiotic fraction and carrier matrix on formulation performance. These results support further investigation of these systems as topical formulation platforms; however, storage stability, release behavior, compositional characterization, and skin-relevant safety and performance require further evaluation before dermocosmetic or dermatological applicability can be established.

## 1. Introduction

The skin is the largest organ of the human body and serves as the first line of defense against physical, chemical, and biological insults while simultaneously regulating immune responses, preventing excessive transepidermal water loss, and maintaining tissue homeostasis [1,2]. As the outermost interface between the host and the external environment, healthy skin harbors a complex and dynamic microbial ecosystem that plays a fundamental role in maintaining barrier integrity and cutaneous immune function. Disturbance of this balanced microbiota or impairment of the skin barrier facilitates colonization by opportunistic microorganisms, including *Staphylococcus aureus*, *Pseudomonas aeruginosa*, and *Candida* spp., thereby increasing susceptibility to acute and chronic skin infections. A major challenge associated with these pathogens is their ability to establish highly organized biofilms, in which microbial cells are embedded within an extracellular polymeric matrix that markedly enhances resistance to antimicrobial agents and host immune defenses. Consequently, biofilm-associated infections frequently delay wound healing, reduce therapeutic efficacy, and contribute to the increasing burden of antimicrobial resistance, emphasizing the urgent need for safe and multifunctional topical alternatives capable of simultaneously protecting the skin barrier and controlling microbial colonization [3]. In recent years, advances in cosmetic science have transformed the concept of topical skin care from products intended solely for cleansing or aesthetic improvement into biologically active formulations capable of supporting skin physiology and microbiome homeostasis. This transition has accelerated the development of dermocosmetic and pharmabiotic products containing functional bioactive compounds with antioxidant, antimicrobial, anti-inflammatory, and barrier-protective properties [4,5,6]. Among available topical dosage forms, gel-serums have attracted considerable attention because of their lightweight texture, rapid absorption, high water content, excellent spreadability, and pleasant sensory characteristics. Furthermore, gel-serum systems enable efficient incorporation of hydrophilic bioactive substances while ensuring homogeneous distribution and prolonged residence time on the skin surface, making them particularly attractive carriers for microbiome-derived active ingredients [7].

Probiotic microorganisms and their derived bioactive products have emerged as promising candidates for next-generation dermocosmetic formulations owing to their broad spectrum of biological activities. Numerous studies have demonstrated that probiotic-derived compounds exhibit antimicrobial, antioxidant, anti-inflammatory, and immunomodulatory effects that may contribute to the maintenance of skin health and restoration of microbial homeostasis [8,9,10]. Nevertheless, the incorporation of viable probiotic microorganisms into topical products remains challenging because their biological activity is highly dependent on storage conditions, formulation composition, and environmental factors. In addition, the presence of live microorganisms may raise safety concerns, particularly for immunocompromised individuals or patients with impaired skin barriers [8]. These limitations have encouraged increasing interest in non-viable probiotic-derived preparations that retain biological activity while offering superior stability and improved formulation compatibility. Among these emerging pharmabiotic ingredients, postbiotics and paraprobiotics have gained particular attention as safe and stable alternatives to conventional probiotics. According to the International Scientific Association for Probiotics and Prebiotics (ISAPP), postbiotics are defined as preparations of inanimate microorganisms and/or their components that confer health benefits to the host [8,11]. These preparations comprise a complex mixture of biologically active compounds, including organic acids, bacteriocins, enzymes, peptides, amino acids, exopolysaccharides, short-chain fatty acids, vitamins, and other microbial metabolites capable of exerting antimicrobial, antioxidant, anti-inflammatory, and immunomodulatory activities [9,10]. In contrast, paraprobiotics consist of metabolically inactive microbial cells that retain structural integrity following controlled inactivation processes. Their biological activity is primarily associated with preserved cell-wall components, including peptidoglycans, lipoteichoic acids, and surface proteins, which interact with host cells and modulate immune responses without requiring microbial viability [12,13]. Compared with conventional probiotics, both postbiotics and paraprobiotics exhibit greater physicochemical stability, longer shelf life, easier manufacturing and storage, and a lower risk of microbial translocation or infection, making them particularly attractive active ingredients for topical pharmabiotic formulations [8,9,11]. Probiotic bacteria, *Lacticaseibacillus casei* is one of the most extensively investigated species because of its remarkable capacity to produce a diverse repertoire of biologically active metabolites with broad therapeutic potential. Previous studies have demonstrated that *L. casei*-derived postbiotics inhibit pathogenic microorganisms through the production of organic acids, bacteriocins, biosurfactants, and other antimicrobial metabolites, while simultaneously reducing oxidative stress and suppressing biofilm formation [9,14]. Similarly, *L. casei* paraprobiotics have been reported to exert beneficial biological effects through preserved cell-wall components, including peptidoglycans, lipoteichoic acids, and surface-associated proteins, which contribute to immune modulation, maintenance of epithelial barrier integrity, and regulation of host–microbe interactions [8,12,13]. Owing to these multifunctional biological properties, *L. casei*-derived postbiotics and paraprobiotics have emerged as promising pharmabiotic ingredients for topical formulations intended to support skin health while minimizing the limitations associated with viable probiotics [15,16,17].

The biological performance of topical pharmabiotic formulations, however, depends not only on the incorporated active ingredient but also on the physicochemical characteristics of the carrier system. An ideal topical vehicle should provide suitable rheological behavior, ease of application, prolonged residence time on the skin surface, and adequate stability while preserving the biological activity of incorporated compounds. Among the available polymeric carriers, Carbopol^®^ and chitosan represent two of the most widely investigated materials because of their distinct physicochemical and functional properties [18,19]. Carbopol^®^ is a synthetic cross-linked poly(acrylic acid) polymer capable of forming transparent, highly viscous hydrogels at relatively low concentrations. Its excellent rheological characteristics, pH stability, high water-retention capacity, and formulation versatility have resulted in widespread application in topical pharmaceutical and cosmetic products [19,20,21,22]. In contrast, chitosan is a naturally derived cationic polysaccharide obtained through the deacetylation of chitin and has attracted considerable interest as a multifunctional biomaterial. Aside from serving as a biodegradable and biocompatible polymeric carrier, chitosan possesses intrinsic antimicrobial, antibiofilm, antioxidant, bioadhesive, and wound-healing properties that may synergistically enhance the biological activity of incorporated pharmabiotic ingredients [23,24]. Electrostatic interactions between positively charged amino groups of chitosan and negatively charged microbial cell surfaces increase membrane permeability, destabilize biofilm architecture, and may facilitate improved antimicrobial efficacy. Moreover, its film-forming ability, high water-holding capacity, and favorable rheological characteristics contribute to prolonged skin retention and enhanced topical performance [23,24,25,26,27,28,29,30]. These complementary characteristics suggest that both Carbopol^®^ and chitosan are attractive polymeric platforms for the development of microbiome-inspired pharmabiotic gel-serums, although their comparative influence on formulation performance has not been comprehensively elucidated. Recent studies have further highlighted the growing interest in postbiotic-based approaches for topical and dermocosmetic applications. Theodorou et al. (2024), in a review of clinical studies involving probiotic- and postbiotic-containing cosmeceuticals, emphasized the increasing use of microbial-derived preparations in skin-care products while also highlighting the need for adequate characterization and standardization [30]. Rusic et al. (2024) evaluated a cosmetic serum containing postbiotics, further demonstrating the growing interest in incorporating microbial-derived bioactives into topical formulations [31]. More recently, Machado et al. (2025), in a scoping review of cutaneous biotic formulations, reported considerable heterogeneity in microbial preparations, formulation strategies, and evaluated outcomes, which limits direct comparison among available studies [32]. Collectively, these recent studies demonstrate the growing potential of postbiotic-based topical formulations but also reveal important limitations in the current literature, particularly the lack of standardized microbial preparations, heterogeneous formulation approaches, and limited systematic evaluation of how carrier systems influence the performance of incorporated microbial-derived ingredients. Despite these advances, comparatively little attention has been given to the influence of the polymeric carrier on the physicochemical and biological performance of postbiotic and paraprobiotic preparations. In particular, direct comparisons of postbiotic and paraprobiotic fractions derived from the same microbial source and incorporated into different polymeric gel matrices remain limited. Addressing this gap may provide further insight into how both the pharmabiotic fraction and the carrier matrix contribute to overall formulation performance.

To address this gap, the present study aimed to develop multifunctional pharmabiotic gel-serums containing *Lacticaseibacillus casei*-derived postbiotic and paraprobiotic fractions using Carbopol^®^ and chitosan as alternative polymeric delivery systems. The developed formulations were characterized in terms of macroscopic appearance, pH, spreadability, viscosity, and rheological properties. Their antimicrobial activity was subsequently evaluated against *Bacillus subtilis*, *Staphylococcus aureus*, *Pseudomonas aeruginosa*, *Candida albicans*, and *Candida krusei*. In addition, minimum inhibitory concentrations, effects on preformed *S. aureus* and *P. aeruginosa* biofilm biomass, and DPPH radical-scavenging activity were investigated. By evaluating both the pharmabiotic fraction (postbiotic and paraprobiotic) and the polymeric carrier (Carbopol^®^ and chitosan), this study provides an integrated framework for assessing their influence on the physicochemical, rheological, and biological performance of the developed gel-serums.

## 2. Results and Discussion

### 2.1. Selection of Polymer Concentrations 

Preliminary formulation screening demonstrated concentration-dependent differences in the macroscopic consistency and handling characteristics of both polymeric systems. For the Carbopol^®^ system, concentrations below 0.25% (*w*/*w*) provided progressively lower structural consistency, whereas 0.30% (*w*/*w*) produced a more consistent system with less favorable handling and spreading characteristics. Carbopol^®^ at 0.25% (*w*/*w*) provided a homogeneous gel-serum with a suitable balance between structural consistency and ease of spreading. For the chitosan system, lower concentrations provided insufficient consistency, whereas concentrations above 0.30% (*w*/*w*) resulted in progressively thicker systems with less favorable spreading characteristics. Accordingly, 0.30% (*w*/*w*) chitosan was selected. Based on the preliminary screening, 0.25% (*w*/*w*) Carbopol^®^ and 0.30% (*w*/*w*) chitosan were selected for preparation of the final formulations (Table 1).

### 2.2. Development of Carbopol^®^-Based Pharmabiotic Gel-Serums

Carbopol^®^-based pharmabiotic gel-serum formulations were successfully developed using Carbopol^®^ 940 as the gelling polymer. Higher Carbopol^®^ concentrations resulted in formulations with excessive viscosity and reduced spreadability, whereas lower concentrations failed to produce an adequately structured gel network. Based on these preliminary observations, a Carbopol^®^ 940 concentration of 0.25% (*w*/*w*) was selected as the optimum concentration for subsequent formulation studies. Following complete hydration of the polymer, sorbitol, polyethylene glycol 400 (PEG 400), urea, sodium salicylate, ammonium benzoate, and Polysorbate 20 were sequentially incorporated into the formulation. Gel formation was achieved by controlled neutralization with triethanolamine (TEA), producing a transparent and homogeneous gel-serum base suitable for topical application. Postbiotic and paraprobiotic fractions were subsequently incorporated into the blank formulation. The paraprobiotic fraction was confirmed to contain no culturable viable cells, as no colony formation was observed on either MRS agar or Nutrient Agar (NA) following incubation under the specified conditions. Based on preliminary formulation studies, the postbiotic fraction was incorporated at 5% (*w*/*w*), corresponding to 5 g of lyophilized postbiotic material per 100 g of final formulation, whereas the paraprobiotic fraction was incorporated at 1% (*w*/*w*), corresponding to 1 g of lyophilized paraprobiotic material per 100 g of final formulation. These loading levels were selected based on preliminary formulation compatibility and dispersion studies. After incorporation of the pharmabiotic fractions, the formulations were gently mixed until complete and homogeneous dispersion of the active components was achieved without visible phase separation or particle aggregation. Three Carbopol^®^-based formulations were prepared for further characterization and biological evaluation: a blank formulation (CA-C-Blank), a postbiotic-loaded formulation (CA-C-PB), and a paraprobiotic-loaded formulation (CA-C-PPB). The composition of the polymeric base remained identical among all formulations, while only the incorporated pharmabiotic fraction was varied to enable comparative evaluation of postbiotic and paraprobiotic incorporation. The qualitative and quantitative composition of the Carbopol^®^-based pharmabiotic gel-serums is presented in Table 2**.**

### 2.3. Development of Chitosan-Based Pharmabiotic Gel-Serums

Chitosan-based pharmabiotic gel-serums were successfully developed using medium molecular weight (MMW) chitosan as the natural polymeric carrier. Preliminary formulation studies were conducted to optimize the polymer concentration and obtain gel-serums with suitable consistency and topical applicability. Higher chitosan concentrations produced formulations with excessive viscosity, whereas lower concentrations resulted in insufficient gel formation. Based on these preliminary observations, a chitosan concentration of 0.30% (*w*/*w*) was selected for subsequent formulation development. Citric acid monohydrate was used to solubilize chitosan and establish the acidic environment required for polymer hydration. A concentration of 0.30% (*w*/*w*) was found to be sufficient for complete dissolution of the polymer and the preparation of homogeneous gel-serum formulations. Following complete hydration of chitosan, sorbitol, polyethylene glycol 400 (PEG 400), urea, sodium salicylate, ammonium benzoate, and Polysorbate 20 were sequentially incorporated into the formulation to prepare the blank gel base. The resulting formulations were homogeneous and exhibited no visible phase separation during preparation. The postbiotic and paraprobiotic fractions were subsequently incorporated into the blank formulation at concentrations of 5% (*w*/*w*) and 1% (*w*/*w*), respectively, corresponding to 5 g of lyophilized postbiotic material and 1 g of lyophilized paraprobiotic material per 100 g of final formulation. After incorporation, all formulations were mixed under gentle stirring until uniform distribution of the pharmabiotic fractions was achieved. Three chitosan-based formulations were prepared for further physicochemical characterization and biological evaluation, including a blank formulation (CA-CS-Blank), a postbiotic-loaded formulation (CA-CS-PB), and a paraprobiotic-loaded formulation (CA-CS-PPB). The composition of the polymeric base remained identical in all formulations, while only the incorporated pharmabiotic fraction differed among the experimental groups. The composition of the developed chitosan-based pharmabiotic gel-serums is presented in Table 3.

### 2.4. Rheological Characterization of Pharmabiotic Gel-Serum Formulations

**Figure 1:**
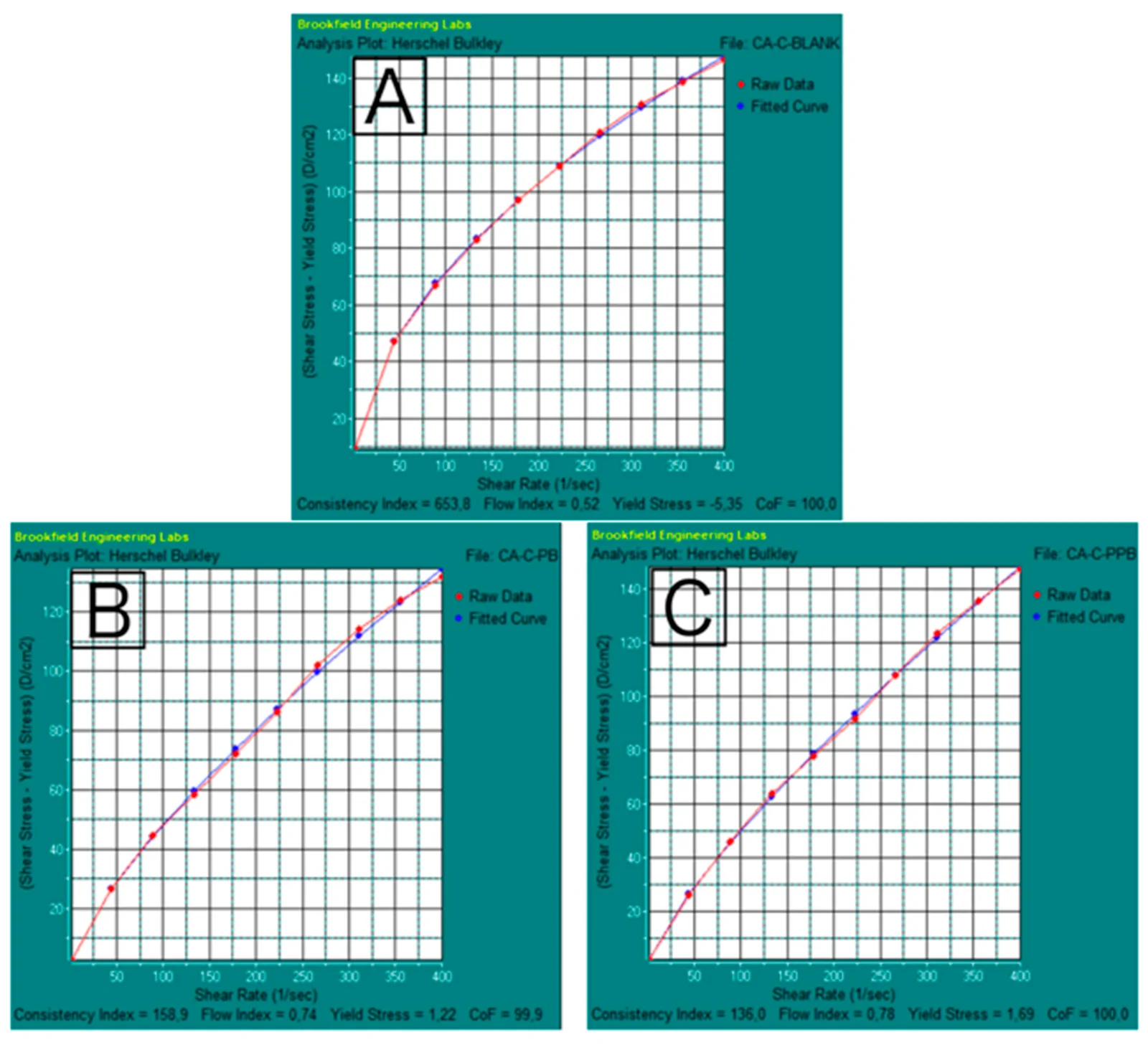
Herschel–Bulkley rheological profiles of Carbopol^®^-based pharmabiotic gel-serum formulations: (**A**) blank formulation (CA-C-Blank), (**B**) postbiotic-loaded formulation (CA-C-PB), and (**C**) paraprobiotic-loaded formulation (CA-C-PPB). Red points represent the experimental data, while blue lines represent the fitted Herschel–Bulkley model.

**Figure 2:**
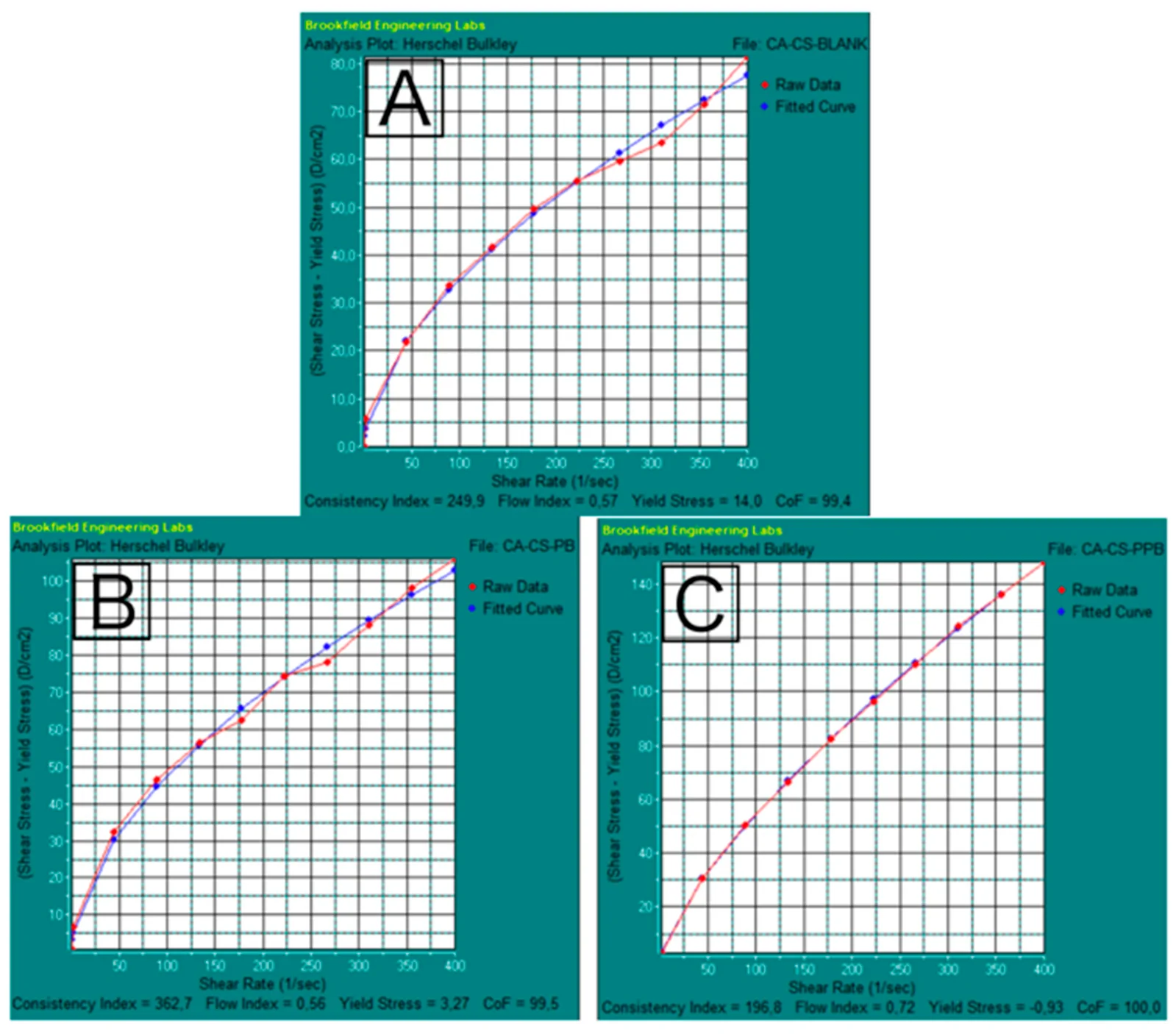
Herschel–Bulkley rheological profiles of chitosan-based pharmabiotic gel-serum formulations: (**A**) blank formulation (CA-CS-Blank), (**B**) postbiotic-loaded formulation (CA-CS-PB), and (**C**) paraprobiotic-loaded formulation (CA-CS-PPB). Red points represent the experimental data, while blue lines represent the fitted Herschel–Bulkley model.

The rheological behavior of the developed Carbopol^®^- and chitosan-based pharmabiotic gel-serum formulations was evaluated using the Herschel–Bulkley model. The yield stress (τ_0_), consistency index (K), flow-behavior index (*n*), coefficient of fit (CoF), and coefficient of determination (R^2^) were determined for each formulation. The experimental flow curves and the corresponding fitted models are presented in Figure 1 and Figure 2, while the calculated rheological parameters are summarized in Table 4. A summary of the rheological characteristics of each formulation is provided in Table 5.

A high degree of agreement was observed between the experimental data and the Herschel–Bulkley model for all formulations. The coefficient of fit (CoF) values ranged from 99.4% to 100.0%, while the corresponding R^2^ values were 0.994–1.000, indicating excellent agreement between the experimental and model-fitted data (Table 3). The calculated flow-behavior index (n) ranged from 0.52 to 0.78 for all formulations (Table 3). Since all n values were below 1.0, all developed formulations exhibited pseudoplastic (shear-thinning) flow behavior. Apparent viscosity values were additionally estimated from the fitted Herschel–Bulkley model at representative shear rates of 10 and 100 s^−1^. At 10 s^−1^, the predicted apparent viscosity ranged from 82.12 to 215.96 Pa·s, whereas at 100 s^−1^ it ranged from 34.64 to 71.63 Pa·s. In all formulations, apparent viscosity decreased with increasing shear rate, further supporting the shear-thinning behavior indicated by the flow-behavior indices. Among the Carbopol^®^-based formulations, CA-C-Blank exhibited the highest predicted apparent viscosity at both shear rates. Among the chitosan-based formulations, CA-CS-PB showed the highest predicted apparent viscosity at 10 s^−1^, whereas CA-CS-PPB exhibited the highest value at 100 s^−1^. Among the Carbopol^®^-based formulations, the blank formulation (CA-C-Blank) exhibited the highest consistency index (K = 653.8 Pa·s^n^) and the lowest flow-behavior index (n = 0.52). Incorporation of the postbiotic fraction decreased the consistency index to 158.9 Pa·s^n^ and increased the flow-behavior index to 0.74, whereas the paraprobiotic-loaded formulation showed a consistency index of 136.0 Pa·s^n^ with a flow-behavior index of 0.78. Yield stress values for the Carbopol^®^-based formulations ranged from −5.35 to 1.69 Pa (Table 3). For the chitosan-based formulations, the blank gel-serum (CA-CS-Blank) exhibited a consistency index of 249.9 Pa·s^n^, a flow-behavior index of 0.57, and the highest yield stress among all formulations (14.00 Pa). The postbiotic-loaded formulation (CA-CS-PB) presented the highest consistency index among the chitosan formulations (362.7 Pa·s^n^) with a flow-behavior index of 0.56, whereas the paraprobiotic-loaded formulation (CA-CS-PPB) exhibited a consistency index of 196.8 Pa·s^n^, a flow-behavior index of 0.72, and a yield stress of −0.93 Pa (Table 3). The negative fitted yield-stress values obtained for CA-C-Blank (−5.35 Pa) and CA-CS-PPB (−0.93 Pa) were interpreted as indicating negligible or near-zero apparent yield stress. These negative numerical values were considered mathematical outputs of the Herschel–Bulkley fitting and were not assigned a physical interpretation. All developed gel-serum formulations were successfully fitted to the Herschel–Bulkley model and demonstrated pseudoplastic flow behavior. The rheological characteristics of each formulation are summarized in Table 4.

All formulations maintained pseudoplastic (shear-thinning) flow behavior despite changes in consistency index and apparent viscosity following the incorporation of PB and PPB. The decrease in apparent viscosity with increasing shear rate is advantageous for topical application because the formulations become less resistant to flow under the shear generated during dispensing and spreading on the skin. This behavior may facilitate dispensing from the container and subsequent spreading over the application surface. The observed spreadability results further support the suitability of the developed gel-serums for topical application, although differences in consistency between the Carbopol^®^- and chitosan-based systems resulted in different spreading characteristics. At lower shear conditions, the comparatively higher apparent viscosity of the formulations may contribute to maintaining the formulation at the application site after spreading; however, skin retention was not directly measured in the present study. Therefore, the rheological findings support favorable dispensing and spreading characteristics, whereas conclusions regarding prolonged skin retention require further direct evaluation. Thixotropic recovery was also not specifically evaluated; consequently, structural recovery after removal of shear cannot be inferred from the present data [33,34].

### 2.5. pH of Pharmabiotic Gel-Serum Formulations

The pH values of the developed Carbopol^®^- and chitosan-based pharmabiotic gel-serum formulations are presented in Table 5. The pH values ranged from 4.22 ± 0.01 to 5.66 ± 0.01, with low standard deviations across all formulations, indicating good measurement reproducibility. Among the Carbopol^®^-based formulations, the blank formulation (CA-C-Blank) exhibited a pH of 4.40 ± 0.02. Incorporation of the postbiotic fraction resulted in a pH of 4.23 ± 0.05, whereas the paraprobiotic-loaded formulation (CA-C-PPB) exhibited the highest pH within the Carbopol^®^ group (5.66 ± 0.01). For the chitosan-based formulations, the blank formulation (CA-CS-Blank) showed a pH of 5.51 ± 0.04. The postbiotic-loaded formulation (CA-CS-PB) exhibited the lowest pH among all developed formulations (4.22 ± 0.01), while the paraprobiotic-loaded formulation (CA-CS-PPB) showed a pH of 5.64 ± 0.03. Comparison of the two polymeric systems revealed that the postbiotic-loaded formulations exhibited nearly identical pH values (4.23 ± 0.05 for CA-C-PB and 4.22 ± 0.01 for CA-CS-PB). Similarly, the paraprobiotic-loaded formulations demonstrated comparable pH values, measuring 5.66 ± 0.01 for CA-C-PPB and 5.64 ± 0.03 for CA-CS-PPB. All developed pharmabiotic gel-serums exhibited pH values within a mildly acidic range. The measured pH values and corresponding standard deviations are presented in Table 6.

The measured pH values ranged from 4.22 to 5.66, remaining within the mildly acidic range compatible with the physiological skin surface. Maintenance of this acidic environment is important for epidermal barrier integrity, antimicrobial defense, and preservation of the resident skin microbiota [35,36] Likewise, all formulations demonstrated satisfactory spreadability and serum-like consistency. Carbopol^®^ formulations exhibited greater spreading areas than the corresponding chitosan formulations, whereas none of the formulations showed phase separation, precipitation, or other signs of physical instability. Collectively, these physicochemical characteristics suggest that both carrier systems are suitable for topical dermocosmetic applications.

### 2.6. Spreadability of Pharmabiotic Gel-Serum Formulations

The spreadability of the developed pharmabiotic gel-serum formulations was evaluated by measuring the spreading diameters along two perpendicular axes (D_1_ and D_2_), calculating the mean spreading diameter, and determining the corresponding spreading area. The results are summarized in Table 7. Among the Carbopol^®^-based formulations, the blank formulation (CA-C-Blank) exhibited a mean spreading diameter of 18.35 mm, corresponding to a spreading area of 264.4 mm^2^. The postbiotic-loaded formulation (CA-C-PB) showed an increased mean spreading diameter of 19.50 mm and a spreading area of 298.6 mm^2^, whereas the paraprobiotic-loaded formulation (CA-C-PPB) demonstrated the highest spreadability within this group, with a mean spreading diameter of 20.35 mm and a spreading area of 325.3 mm^2^. For the chitosan-based formulations, the blank formulation (CA-CS-Blank) exhibited a mean spreading diameter of 16.35 mm and a spreading area of 209.9 mm^2^. The postbiotic-loaded formulation (CA-CS-PB) showed the lowest spreadability among all formulations, with a mean spreading diameter of 15.25 mm and a spreading area of 182.7 mm^2^. In contrast, the paraprobiotic-loaded formulation (CA-CS-PPB) exhibited a mean spreading diameter of 17.20 mm and a spreading area of 232.2 mm^2^. Comparison of the two polymeric systems showed that the Carbopol^®^-based formulations exhibited larger spreading diameters and spreading areas than the corresponding chitosan-based formulations. Among all developed gel-serums, CA-C-PPB demonstrated the greatest spreadability, whereas CA-CS-PB exhibited the lowest spreading area.

### 2.7. Macroscopic Characteristics of Pharmabiotic Gel-Serum Formulations

The macroscopic characteristics of the developed pharmabiotic gel-serum formulations were evaluated in terms of color, appearance, homogeneity, phase separation, precipitation, and flow properties. The results are summarized in Table 8. The blank formulations exhibited a colorless appearance, with the chitosan-based formulation (CA-CS-Blank) presenting a slightly opalescent appearance and the Carbopol^®^-based formulation (CA-C-Blank) appearing transparent. Both blank formulations were homogeneous and showed no visible phase separation or precipitation. Following incorporation of the postbiotic fraction, both Carbopol^®^- and chitosan-based gel-serums developed a brown coloration while maintaining a homogeneous appearance. In contrast, the paraprobiotic-loaded formulations exhibited a cream–white color with a slightly opaque appearance in both polymeric systems. During the initial post-preparation evaluation, all formulations exhibited a homogeneous macroscopic appearance, with no visible particle aggregation, precipitation, or phase separation. These observations indicate satisfactory initial physical compatibility of the formulation components but should not be interpreted as evidence of long-term storage stability. Furthermore, all formulations exhibited homogeneous serum-like consistency throughout the macroscopic evaluation.

The development of effective topical pharmabiotic formulations requires not only biologically active ingredients but also an appropriate carrier system capable of preserving their stability and enhancing their biological performance. In the present study, *Lacticaseibacillus casei*-derived postbiotic and paraprobiotic fractions were successfully incorporated into Carbopol^®^- and chitosan-based gel-serum formulations. All formulations exhibited a homogeneous appearance immediately after preparation, with no visible phase separation or precipitation during the initial macroscopic evaluation. These findings demonstrate the suitability of both Carbopol^®^ and chitosan as carrier systems for microbiome-derived topical formulations.

### 2.8. Antimicrobial Activity of Pharmabiotic Gel-Serum Formulations

**Figure 3:**
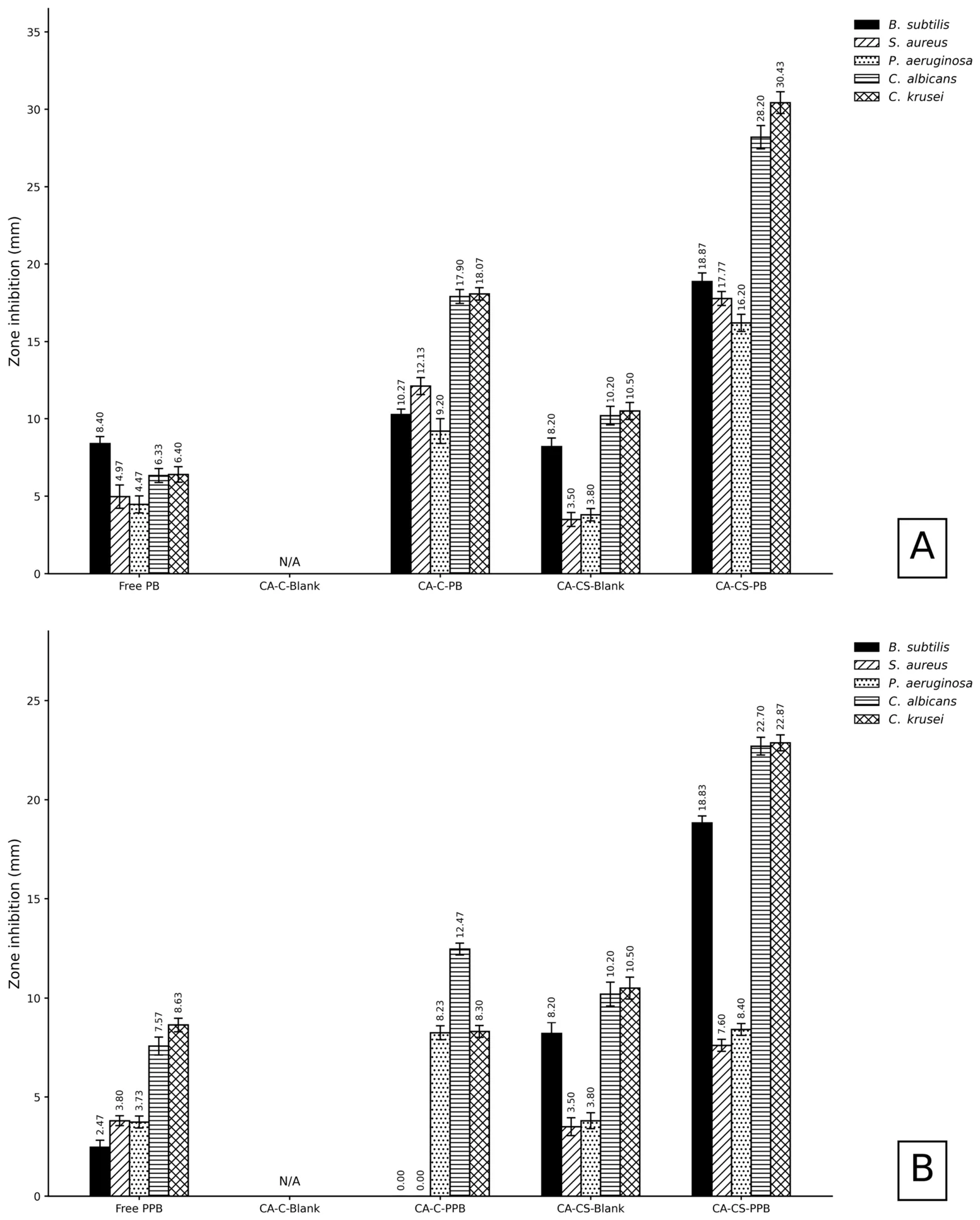
Antimicrobial activity of the developed pharmabiotic gel-serum formulations determined by the agar well-diffusion assay. (**A**) Postbiotic-loaded Carbopol^®^ (CA-C-PB) and chitosan-based (CA-CS-PB) pharmabiotic gel-serum formulations. (**B**) Paraprobiotic-loaded Carbopol^®^ (CA-C-PPB) and chitosan-based (CA-CS-PPB) pharmabiotic gel-serum formulations. Data are presented as mean ± standard deviation (SD) (n = 3). Statistical differences among groups were analyzed using one-way ANOVA followed by Tukey’s multiple comparison test (*p* < 0.0001).

The antimicrobial activities of free *Lacticaseibacillus casei*-derived postbiotic (PB) and paraprobiotic (PPB) fractions and the corresponding complete Carbopol^®^-based (CA-C-PB and CA-C-PPB) and chitosan-based (CA-CS-PB and CA-CS-PPB) pharmabiotic gel-serum formulations were comparatively evaluated. Blank Carbopol^®^ (CA-C-Blank) and chitosan (CA-CS-Blank) formulations were included as vehicle controls to distinguish the contribution of the polymeric formulation matrix from that of the incorporated pharmabiotic fractions. The agar diffusion results are presented in Figure 3, whereas the minimum inhibitory concentration (MIC) values are summarized in Table 9 and Table 10. Statistical analysis of the inhibition zone diameters revealed significant differences among the experimental groups (*p* < 0.0001).

The postbiotic fraction exhibited antimicrobial activity against all tested microorganisms, producing inhibition zone diameters ranging from 4.47 to 8.40 mm. The largest inhibition zone was observed against *Bacillus subtilis* (8.40 mm), followed by *Candida krusei* (6.40 mm), *Candida albicans* (6.33 mm), *Staphylococcus aureus* (4.97 mm), and *Pseudomonas aeruginosa* (4.47 mm) (Figure 3). Following incorporation into the Carbopol^®^-based gel-serum (CA-C-PB), inhibition zone diameters increased for all tested microorganisms. The largest inhibition zones were observed against *C. krusei* (18.07 mm) and *C. albicans* (17.90 mm). Similarly, the chitosan-based postbiotic formulation (CA-CS-PB) demonstrated broad antimicrobial activity, with inhibition zones of 19.93 mm against *C. krusei*, 18.00 mm against *C. albicans*, and 12.40 mm against *P. aeruginosa* (Figure 3). The MIC values of the postbiotic formulations are presented in Table 8. However, inhibition-zone diameters obtained by agar diffusion should be interpreted cautiously because they reflect not only antimicrobial activity but also the ability of active constituents to diffuse from the formulation into the agar matrix. Differences in polymer composition, viscosity, and interactions between the incorporated fractions and the polymeric network may therefore influence the apparent zone diameter. Consequently, direct comparison of inhibition-zone sizes between Carbopol^®^- and chitosan-based formulations cannot be considered an unequivocal measure of differences in intrinsic antimicrobial potency. For this reason, the agar diffusion findings were interpreted together with the MIC results and the corresponding blank formulation controls. The purified postbiotic fraction exhibited MIC values of 2500 μg/mL against *B. subtilis* and *P. aeruginosa*, and 5000 μg/mL against *S. aureus*, *C. albicans*, and *C. krusei*. Both Carbopol^®^-based (CA-C-PB) and chitosan-based (CA-CS-PB) formulations exhibited MIC values of 3125 μg/mL against the bacterial strains, whereas lower MIC values (1156 μg/mL) were obtained against *C. albicans* and *C. krusei* (Table 8). Compared with the postbiotic fraction, the purified paraprobiotic exhibited lower antimicrobial activity. Inhibition zone diameters ranged from 2.47 to 8.63 mm, with the largest inhibition zone observed against *C. krusei* and the smallest against *B. subtilis* (Figure 3B). The Carbopol^®^-based paraprobiotic formulation (CA-C-PPB) did not produce detectable inhibition zones against *B. subtilis* or *S. aureus*, whereas inhibition was observed against *P. aeruginosa*, *C. albicans*, and *C. krusei*. In contrast, the chitosan-based formulation (CA-CS-PPB) inhibited all tested microorganisms, producing inhibition zones of 10.63 mm against *B. subtilis*, 12.50 mm against *C. albicans*, and 12.37 mm against *C. krusei* (Figure 3B). The MIC values of the paraprobiotic formulations are presented in Table 9. The purified paraprobiotic exhibited MIC values of 10,000 μg/mL against the bacterial strains and 5000 μg/mL against the *Candida* species. The Carbopol^®^-based formulation (CA-C-PPB) showed no detectable inhibitory activity against *B. subtilis* or *S. aureus*, while MIC values of 3125 μg/mL were obtained against *P. aeruginosa*, *C. albicans*, and *C. krusei*. The chitosan-based formulation (CA-CS-PPB) exhibited MIC values of 6250 μg/mL against *B. subtilis* and 3125 μg/mL against *S. aureus*, *P. aeruginosa*, *C. albicans*, and *C. krusei* (Table 9). The postbiotic-loaded formulations (CA-C-PB and CA-CS-PB) exhibited greater antimicrobial activity than the corresponding paraprobiotic-loaded formulations (CA-C-PPB and CA-CS-PPB) in both agar diffusion and MIC assays. Among all tested formulations, the largest inhibition zones were observed for the chitosan-based postbiotic gel-serum (CA-CS-PB), whereas the lowest MIC values were obtained against *C. albicans* and *C. krusei* (1156 μg/mL) in both postbiotic-loaded formulations. The comparatively pronounced antifungal activity observed for CA-CS-PB may reflect contributions from both the incorporated postbiotic fraction and the chitosan-based formulation matrix. Chitosan is known to possess intrinsic antimicrobial properties, and the measurable activity of the corresponding blank chitosan formulation supports a contribution of the vehicle itself to the overall biological response. Nevertheless, the greater activity of the complete CA-CS-PB formulation relative to the blank vehicle indicates that the observed response cannot be attributed solely to the chitosan-based matrix. Interactions among chitosan, formulation excipients, and bioactive constituents of the postbiotic fraction may also influence the overall antimicrobial performance. However, the present study did not directly evaluate release kinetics or specific interactions between chitosan and postbiotic constituents. Therefore, enhanced release or synergistic interactions cannot be established as mechanisms underlying the greater antifungal activity. Further release studies and appropriately designed interaction assays would be required to distinguish between additive, synergistic, and carrier-mediated effects.

The biological evaluation demonstrated that postbiotic-containing formulations generally exhibited stronger antimicrobial activity than the corresponding paraprobiotic-containing formulations. This finding is consistent with previous reports indicating that postbiotic preparations may contain extracellular metabolites, including organic acids, bacteriocins, antimicrobial peptides, biosurfactants, and other bioactive molecules capable of directly affecting pathogenic microorganisms [8,9,14]. Importantly, the inclusion of blank Carbopol^®^ and chitosan formulations as vehicle controls allowed the antimicrobial responses of the complete formulations to be interpreted in the context of both the incorporated pharmabiotic fraction and the formulation matrix. Therefore, differences observed between the free PB/PPB fractions and the complete gel-serums cannot be attributed exclusively to the microbial-derived fractions, as the polymeric carriers and other formulation components may also contribute to the observed antimicrobial effects. Among the developed formulations, CA-CS-PB showed the largest inhibition zones against several tested microorganisms. This finding may partly reflect the intrinsic antimicrobial properties of chitosan, whose positively charged amino groups can interact with negatively charged microbial cell surfaces and alter membrane integrity [23,24]. However, because agar inhibition-zone measurements are also influenced by the diffusion characteristics of the tested formulations, differences in zone diameter should not be interpreted solely as differences in intrinsic antimicrobial potency. The findings indicate that the observed antimicrobial performance reflects the combined contribution of the incorporated pharmabiotic fraction and the formulation matrix.

The blank Carbopol^®^ (CA-C-Blank) and chitosan (CA-CS-Blank) formulations exhibited detectable antimicrobial activity, demonstrating that the carrier systems themselves contributed to the overall antimicrobial performance of the developed gel-serums. This activity should be attributed to the complete blank formulation rather than exclusively to the polymer, since both vehicle systems contained excipients with potential antimicrobial effects. Among the blank formulations, CA-CS-Blank showed measurable inhibition against all tested microorganisms in the agar well-diffusion assay. Inhibition-zone diameters of approximately 8.20, 3.50, 3.80, 10.20, and 10.50 mm were observed against *Bacillus subtilis*, *Staphylococcus aureus*, *Pseudomonas aeruginosa*, *Candida albicans*, and *Candida krusei*, respectively. The greatest inhibition was observed against the two *Candida* species, whereas comparatively smaller zones were obtained against *S. aureus* and *P. aeruginosa*. These findings indicate that the chitosan-based vehicle possessed an intrinsic antimicrobial contribution and that the magnitude of this effect was microorganism-dependent. The antimicrobial activity of CA-CS-Blank is consistent with the known antimicrobial properties of chitosan. However, the observed activity cannot be attributed solely to chitosan because the blank formulation also contained other formulation components, including sodium salicylate and ammonium benzoate, which may contribute to microbial growth inhibition. Thus, the inhibition produced by CA-CS-Blank most likely represents the combined effect of the polymeric matrix and the associated excipients. The relatively pronounced activity against *C. albicans* and *C. krusei* further indicates that the contribution of the blank vehicle should be considered particularly carefully when interpreting the antifungal activity of the corresponding chitosan-based PB- and PPB-containing formulations. CA-C-Blank was similarly included to assess the contribution of the Carbopol^®^-based carrier and its excipients. Any antimicrobial activity detected for this formulation was interpreted as vehicle-associated activity rather than as an intrinsic effect of Carbopol^®^ alone, particularly because the formulation contained sodium salicylate and ammonium benzoate in addition to other excipients. The blank control therefore provided an essential reference for determining the extent to which antimicrobial activity of the corresponding Carbopol^®^-based complete formulations could be associated with the incorporated pharmabiotic fractions. The blank formulations were also included as independent vehicle controls in the broth microdilution assay. Their MIC profiles were considered separately from those of the PB- and PPB-containing formulations rather than being mathematically subtracted from the MIC values of the complete formulations. This distinction is important because MIC represents the lowest tested concentration preventing detectable microbial growth and is therefore not an additive parameter from which a “net MIC” can be calculated by subtraction. Consequently, antimicrobial activity associated with CA-C-Blank and CA-CS-Blank was used as a reference for interpreting the corresponding complete formulations. These findings demonstrate that the polymeric vehicles were not biologically inert under the experimental conditions. The contribution was particularly evident for the chitosan-based blank formulation and varied according to the tested microorganism. Therefore, inclusion of CA-C-Blank and CA-CS-Blank as independent controls was essential for distinguishing vehicle-associated antimicrobial effects from the overall antimicrobial performance of the PB- and PPB-containing gel-serums.

### 2.9. Antibiofilm Activity of Pharmabiotic Gel-Serum Formulations

**Figure 4:**
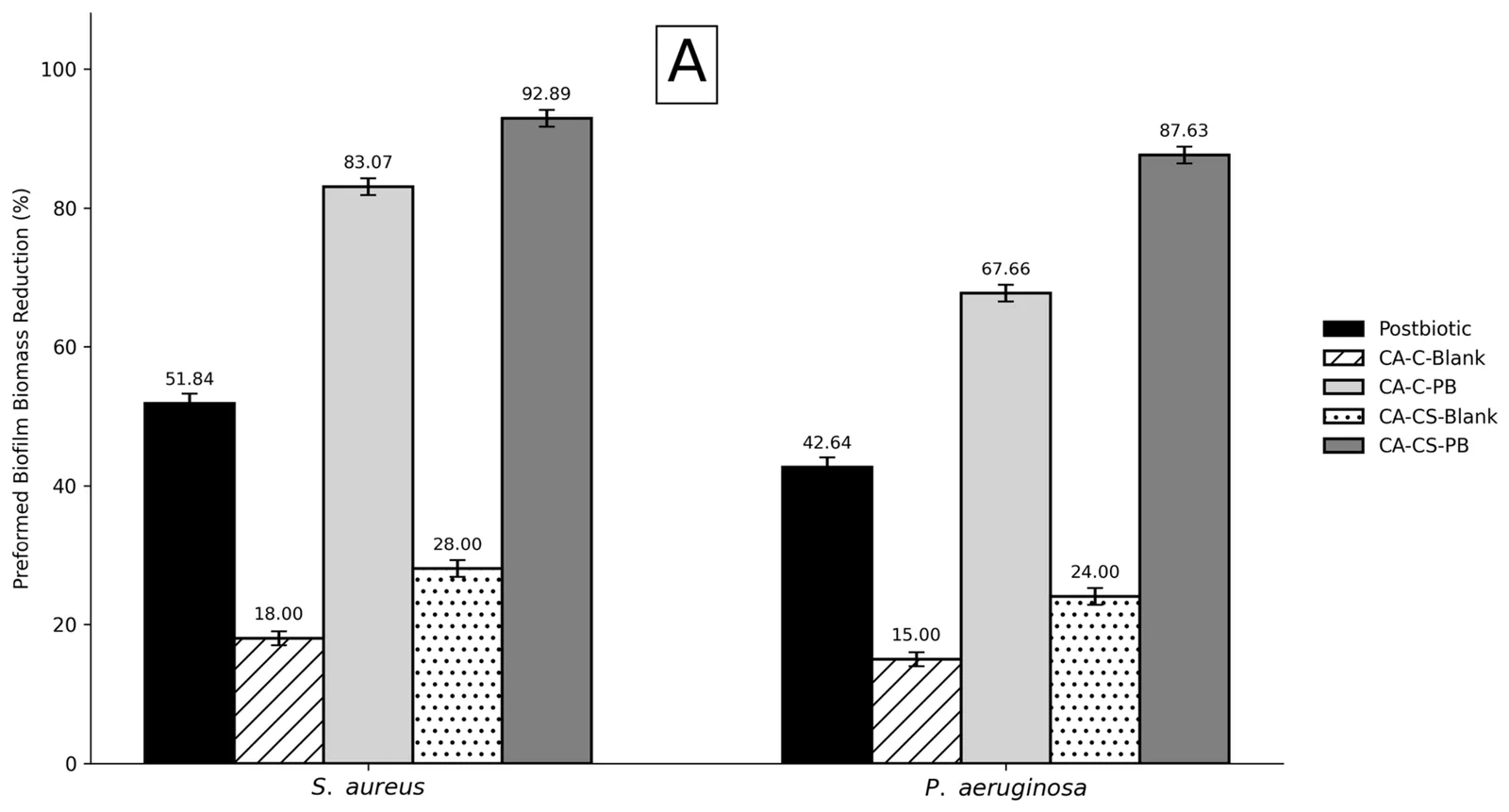

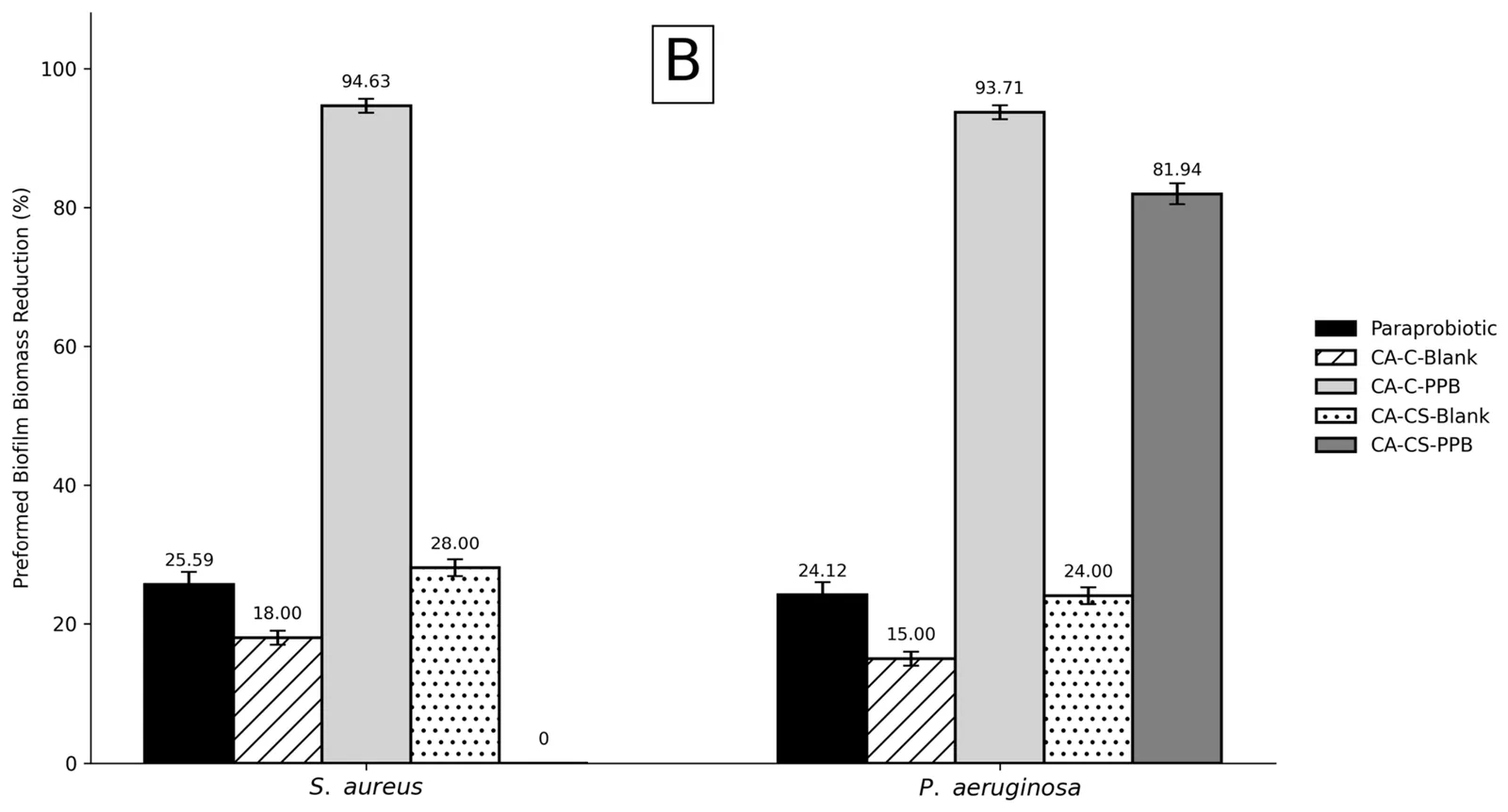
Antibiofilm activity of the developed pharmabiotic gel-serum formulations against *Staphylococcus aureus* ATCC 29213 and *Pseudomonas aeruginosa* ATCC 27853. (**A**) Postbiotic fraction, Carbopol^®^-based postbiotic gel-serum (CA-C-PB), and chitosan-based postbiotic gel-serum (CA-CS-PB). (**B**) Paraprobiotic fraction, Carbopol^®^-based paraprobiotic gel-serum (CA-C-PPB), and chitosan-based paraprobiotic gel-serum (CA-CS-PPB). Data are presented as mean ± standard deviation (SD) (n = 3). Statistical significance was determined using one-way ANOVA followed by Tukey’s multiple comparison test (*p* < 0.0001).

The effects of free *Lacticaseibacillus casei*-derived postbiotic (PB) and paraprobiotic (PPB) fractions, blank Carbopol^®^ (CA-C-Blank) and chitosan (CA-CS-Blank) formulations, and the corresponding complete Carbopol^®^-based (CA-C-PB and CA-C-PPB) and chitosan-based (CA-CS-PB and CA-CS-PPB) pharmabiotic gel-serums on preformed *Staphylococcus aureus* ATCC 29213 and *Pseudomonas aeruginosa* ATCC 27853 biofilm biomass were comparatively evaluated using the crystal-violet assay. The results are presented in Figure 4. Statistical analysis revealed significant differences among the experimental groups (*p* < 0.0001). The blank formulations showed measurable effects on preformed biofilm biomass, indicating that the formulation matrices were not biologically inert. CA-C-Blank reduced preformed biofilm biomass by 18.00% against *S. aureus* and 15.00% against *P. aeruginosa*, whereas CA-CS-Blank produced reductions of 28.00% and 24.00%, respectively. The greater reductions observed with CA-CS-Blank may be associated, at least in part, with the intrinsic antimicrobial and biofilm-modulating properties of chitosan. Nevertheless, the effects of the blank formulations cannot be attributed exclusively to Carbopol^®^ or chitosan, because other formulation components may also influence microbial growth, attachment, and retained biofilm biomass. The free postbiotic fraction reduced preformed biofilm biomass by 51.84% against *S. aureus* and 42.64% against *P. aeruginosa*. Incorporation of the postbiotic fraction into the Carbopol^®^-based gel-serum (CA-C-PB) was associated with reductions of 83.07% and 67.66% against *S. aureus* and *P. aeruginosa*, respectively, compared with 18.00% and 15.00% for CA-C-Blank. The chitosan-based postbiotic formulation (CA-CS-PB) produced reductions of 92.89% against *S. aureus* and 87.63% against *P. aeruginosa*, substantially exceeding the corresponding reductions of 28.00% and 24.00% observed with CA-CS-Blank. The free paraprobiotic fraction exhibited lower activity than the free postbiotic fraction, reducing preformed biofilm biomass by 25.59% against *S. aureus* and 24.12% against *P. aeruginosa*. In contrast, the Carbopol^®^-based paraprobiotic formulation (CA-C-PPB) produced pronounced reductions of 94.63% against *S. aureus* and 93.71% against *P. aeruginosa*, compared with 18.00% and 15.00%, respectively, for CA-C-Blank. The chitosan-based paraprobiotic formulation (CA-CS-PPB) reduced preformed *P. aeruginosa* biofilm biomass by 81.94%, considerably exceeding the 24.00% reduction observed with CA-CS-Blank. However, no detectable reduction in preformed *S. aureus* biofilm biomass was observed with CA-CS-PPB, despite the 28.00% reduction observed with the corresponding blank formulation. This finding indicates that the effects of the carrier matrix and the incorporated paraprobiotic fraction were not simply additive under the experimental conditions. The comparison of the free fractions, blank formulations, and complete gel-serums demonstrated that the polymeric vehicles contributed to the observed effects on preformed biofilm biomass. However, the substantially greater reductions produced by CA-C-PB, CA-CS-PB, and CA-C-PPB relative to their corresponding blank formulations indicate that vehicle-associated activity alone did not account for the performance of these complete formulations. Accordingly, the blank formulations were treated as independent vehicle controls rather than mathematically subtracted from the complete formulations, and the observed effects were interpreted as formulation-level responses reflecting the combined influence of the pharmabiotic fraction, polymeric matrix, and formulation excipients. Particularly noteworthy was the pronounced effect of CA-C-PPB on preformed biofilms, resulting in 94.63% and 93.71% reductions in the biomass of *S. aureus* and *P. aeruginosa*, respectively. These values were substantially higher than those observed for the free PPB fraction, which produced reductions of 25.59% and 24.12%, respectively. The corresponding blank Carbopol^®^ formulation was therefore included as a vehicle control to account for the potential contribution of the formulation matrix and excipients. The marked response observed with CA-C-PPB suggests that its effect on preformed biofilm biomass reflects the performance of the complete formulation rather than that of the PPB fraction alone. However, the present data do not establish the mechanism responsible for this pronounced response. Potential interactions among the PPB-derived components, polymeric matrix, and formulation excipients may influence their availability or interaction with the established biofilm, but release behavior and specific component interactions were not directly investigated in the present study. Therefore, these mechanisms require further experimental investigation. Importantly, because treatment was applied after 24 h of biofilm formation, the observed effects represent reductions in preformed biofilm biomass rather than inhibition of initial biofilm formation. Moreover, the crystal-violet assay quantifies total attached biomass and does not distinguish between viable and non-viable cells. Accordingly, the pronounced response observed for CA-C-PPB should not be interpreted as evidence of approximately 94% killing or eradication of biofilm-associated cells. Rather, it indicates a substantial reduction in the amount of biomass remaining attached after treatment. Complementary viability-based assays and microscopy would be required to determine whether this response reflects cell killing, biomass detachment, disruption of the extracellular matrix, or a combination of these effects.

In the present study, the effects on preformed biofilm biomass varied according to both the incorporated pharmabiotic fraction and the polymeric carrier system. Among the postbiotic-containing formulations, CA-CS-PB produced the greatest reduction in preformed biofilm biomass for both *S. aureus* and *P. aeruginosa*, whereas CA-C-PPB showed the greatest reduction among the paraprobiotic-containing formulations. Importantly, the inclusion of blank Carbopol^®^ and chitosan formulations as vehicle controls indicates that the responses of the complete formulations should not be attributed exclusively to the incorporated PB or PPB fractions, since the polymeric matrices and other formulation components may also contribute to the observed effects. Previous studies have reported that postbiotic-derived components may affect microbial adhesion, quorum-sensing-related processes, and extracellular polymeric substances, while chitosan itself can influence biofilm-associated properties through interactions with negatively charged microbial surfaces [9,24]. However, the crystal-violet assay quantifies total attached biofilm biomass rather than viable microbial cells; therefore, the present findings should be interpreted as reductions in preformed biofilm biomass rather than direct evidence of biofilm eradication or killing of biofilm-associated cells.

The crystal-violet assay used in the present study provides a quantitative assessment of total attached biofilm biomass but does not provide direct information on biofilm architecture, extracellular polymeric substance organization, or the spatial distribution and viability of biofilm-associated cells. Therefore, although the observed reductions in preformed biofilm biomass indicate an effect of the formulations on established biofilms, the underlying structural changes cannot be determined from the present assay. Future studies employing confocal laser scanning microscopy or other microscopy-based approaches would be valuable for visualizing changes in biofilm architecture and determining whether the formulations affect extracellular polymeric substances and microbial organization.

### 2.10. DPPH Radical-Scavenging Activity of Pharmabiotic Gel-Serum Formulations

**Figure 5:**
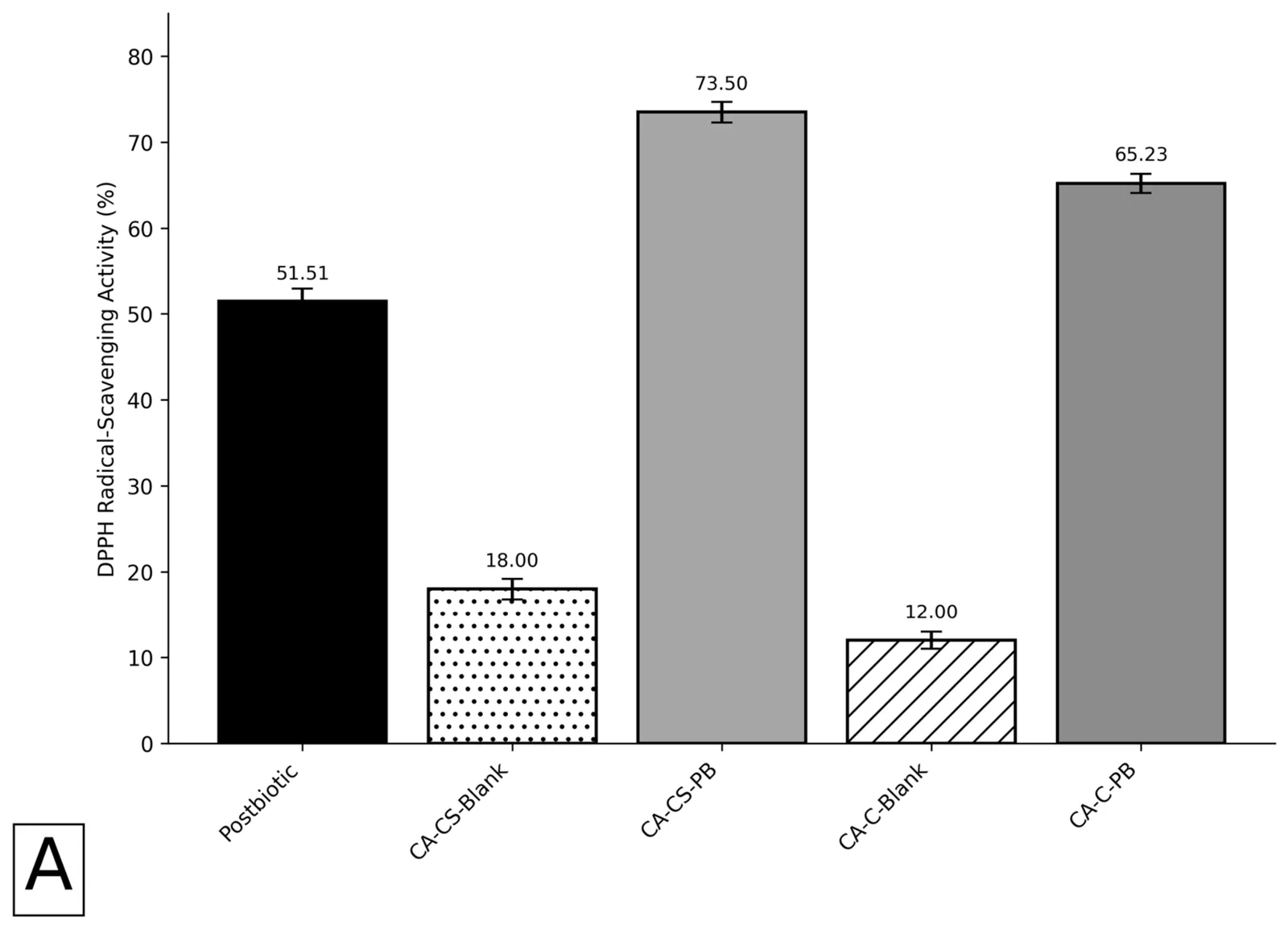

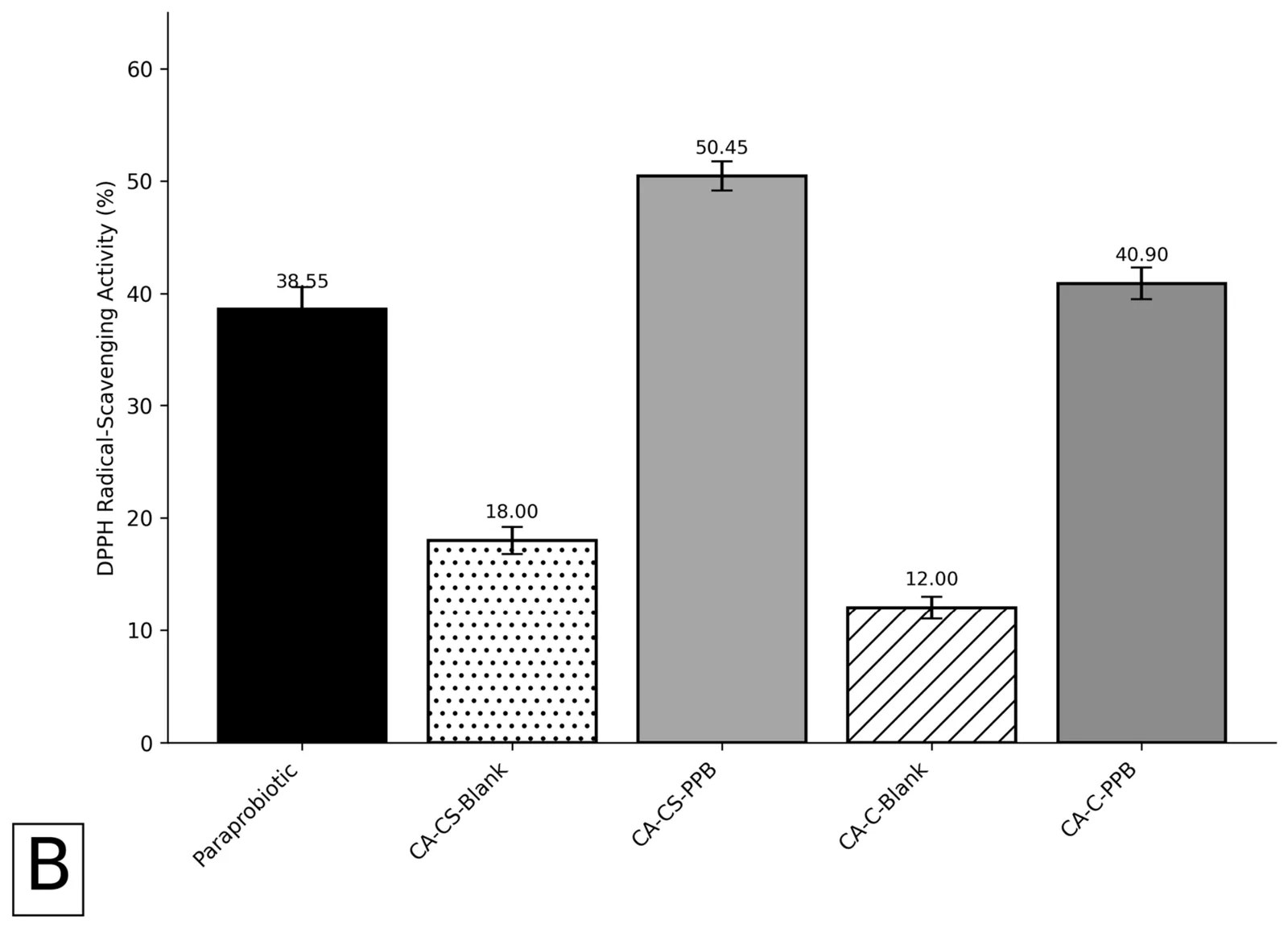
DPPH radical-scavenging activity of the developed pharmabiotic gel-serum formulations determined by the DPPH radical-scavenging assay. (**A**) DPPH radical-scavenging activity of the purified postbiotic fraction and postbiotic-loaded Carbopol^®^ (CA-C-PB) and chitosan-based (CA-CS-PB) pharmabiotic gel-serum formulations. (**B**) DPPH radical-scavenging activity of the purified paraprobiotic fraction and paraprobiotic-loaded Carbopol^®^ (CA-C-PPB) and chitosan-based (CA-CS-PPB) pharmabiotic gel-serum formulations. Data are presented as mean ± standard deviation (SD) (n = 3). Statistical significance was determined using one-way ANOVA followed by Tukey’s multiple comparison test (*p* < 0.0001).

The DPPH radical-scavenging activity of *Lacticaseibacillus casei*-derived postbiotic (PB) and paraprobiotic (PPB) fractions, blank Carbopol^®^ (CA-C-Blank) and chitosan (CA-CS-Blank) formulations, and the corresponding complete Carbopol^®^-based (CA-C-PB and CA-C-PPB) and chitosan-based (CA-CS-PB and CA-CS-PPB) pharmabiotic gel-serums was evaluated using the DPPH free radical-scavenging assay. The results are presented in Figure 5. Statistical analysis demonstrated significant differences among the experimental groups (*p* < 0.0001). The blank formulations exhibited measurable DPPH radical-scavenging activity, indicating that the formulation matrices were not completely inactive in this assay. CA-C-Blank showed a DPPH radical-scavenging activity of 12.00%, whereas CA-CS-Blank exhibited an activity of 18.00%. These responses were interpreted as formulation-level effects rather than being attributed exclusively to Carbopol^®^ or chitosan, since other excipients present in the blank formulations may also interact with the DPPH radical or influence absorbance at the measurement wavelength. The free PB fraction exhibited a DPPH radical-scavenging activity of 51.51%. Incorporation of PB into the Carbopol^®^-based gel-serum (CA-C-PB) resulted in a scavenging activity of 65.23%, compared with 12.00% for CA-C-Blank. The chitosan-based postbiotic formulation (CA-CS-PB) exhibited the highest DPPH radical-scavenging activity among the PB-containing formulations, reaching 73.50%, compared with 18.00% for the corresponding CA-CS-Blank formulation. Thus, although both blank vehicles exhibited some intrinsic response in the DPPH assay, the activities of the complete PB-containing formulations were substantially greater than those of their respective blank controls. The free PPB fraction exhibited a DPPH radical-scavenging activity of 38.55%. CA-C-PPB showed an activity of 40.90%, compared with 12.00% for CA-C-Blank, whereas CA-CS-PPB exhibited a DPPH radical-scavenging activity of 50.45%, compared with 18.00% for CA-CS-Blank. Among the PPB-containing formulations, CA-CS-PPB therefore showed the greatest DPPH radical-scavenging activity. Overall, the PB-containing formulations exhibited greater DPPH radical-scavenging activity than the corresponding PPB-containing formulations, with CA-CS-PB showing the highest activity among all developed gel-serums. Importantly, the activities of the blank formulations were not mathematically subtracted from those of the complete formulations. Instead, CA-C-Blank and CA-CS-Blank were treated as independent vehicle controls and directly compared with their corresponding PB- and PPB-containing formulations. This approach was considered more appropriate because the response of a complete formulation in the DPPH assay may reflect interactions among the polymeric matrix, excipients, and incorporated pharmabiotic fraction and therefore may not be strictly additive. Furthermore, because the DPPH assay measures radical-scavenging capacity under the specific experimental conditions employed, these findings should be interpreted as DPPH radical-scavenging activity rather than as a comprehensive measure of antioxidant activity.

Oxidative stress is closely associated with skin aging, chronic inflammation, and impaired wound healing, making antioxidant activity an important characteristic of dermocosmetic formulations [34]. The present study demonstrated that postbiotic formulations exhibited greater DPPH radical-scavenging activity than the corresponding paraprobiotic formulations. This observation is consistent with previous reports attributing the antioxidant capacity of postbiotics to extracellular metabolites such as antioxidant peptides, exopolysaccharides, organic acids, and other bioactive compounds [14,33]. Furthermore, chitosan-based formulations displayed higher antioxidant activity than Carbopol^®^ formulations, probably reflecting the intrinsic free radical-scavenging capacity of chitosan and its ability to stabilize incorporated bioactive compounds [23,37]. The DPPH assay reflects the free-radical-scavenging capacity of the tested preparations under the experimental conditions employed and does not provide a comprehensive assessment of antioxidant activity. Therefore, the present findings should be interpreted specifically as DPPH radical-scavenging potential. Complementary assays, including ABTS, FRAP, ORAC, and cell-based intracellular ROS measurements, would be valuable in future studies to provide a broader characterization of the antioxidant properties of the developed formulations.

An important aspect of this study is the direct comparison of postbiotic and paraprobiotic fractions incorporated into two different polymeric carrier systems [38,39]. Because PB and PPB were incorporated at different concentrations (5% and 1% *w*/*w*, respectively), direct comparison of their absolute biological activities should be interpreted with caution. The observed differences represent the performance of the final formulations at their selected formulation-specific concentrations and should not be interpreted as an equivalent-dose comparison of the intrinsic biological potency of PB and PPB. Although previous studies have investigated either probiotic-derived metabolites or topical hydrogel carriers individually, comparative evaluations integrating physicochemical characterization with antimicrobial, antibiofilm, and radical-scavenging analyses remain limited. The present findings indicate that the biological performance of the formulations is influenced not only by the pharmabiotic fraction but also by the characteristics of the delivery system, emphasizing the importance of carrier selection during formulation development. The inclusion of blank Carbopol^®^ and chitosan formulations as vehicle controls further demonstrated that the formulation matrices and excipients themselves may contribute to the observed antimicrobial, preformed biofilm biomass reduction, and DPPH radical-scavenging responses. Therefore, the biological effects of the complete formulations were interpreted relative to their corresponding blank controls rather than being attributed exclusively to the incorporated PB or PPB fractions. This study has several limitations. The biological activities were evaluated exclusively under in vitro conditions, and skin-relevant safety and performance assessments were not included in the present experimental design. In particular, cytocompatibility, skin irritation and sensitization, and permeation/penetration studies were not performed. Therefore, the present findings should be interpreted as an initial formulation and in vitro biological characterization and do not establish dermocosmetic or dermatological applicability. Although the PB and PPB preparations were standardized with respect to microbial source, processing conditions, physical form, and incorporation level, detailed compositional profiling was not performed. Future studies employing targeted compositional analyses, including characterization of organic acids, proteins/peptides, carbohydrates, and other microbial-derived constituents, would enable more comprehensive standardization and facilitate mechanistic comparison of the two preparations. In addition, long-term storage stability, release kinetics, cytocompatibility, irritation and sensitization, and ex vivo permeation/penetration should be investigated to further characterize the safety and performance of the developed formulations. The results demonstrate the feasibility of incorporating *Lacticaseibacillus casei*-derived postbiotic and paraprobiotic fractions into Carbopol^®^- and chitosan-based gel-serums while maintaining favorable physicochemical characteristics and exhibiting antimicrobial activity, effects on preformed biofilm biomass, and DPPH radical-scavenging activity. Among the tested complete formulations, the chitosan-based postbiotic gel-serum showed the most balanced overall biological performance, whereas the Carbopol^®^-based paraprobiotic formulation produced particularly pronounced reductions in preformed biofilm biomass. These formulation-level effects should be interpreted in relation to the corresponding blank vehicle controls, which also exhibited measurable biological responses.

## 3. Conclusions

This study demonstrated the feasibility of incorporating *Lacticaseibacillus casei*-derived postbiotic (PB) and paraprobiotic (PPB) fractions into Carbopol^®^- and chitosan-based pharmabiotic gel-serum systems. The developed formulations exhibited homogeneous initial macroscopic characteristics, skin-compatible pH values, pseudoplastic shear-thinning behavior, and formulation-dependent spreadability. The rheological and physicochemical findings further showed that both the pharmabiotic fraction and the polymeric carrier influenced gel-serum characteristics. The formulations also exhibited antimicrobial activity, reductions in preformed biofilm biomass, and DPPH radical-scavenging activity, with the magnitude of these effects varying according to both the incorporated fraction and the polymeric carrier. Among the PB-containing systems, the chitosan-based formulation showed a favorable overall biological performance, whereas the Carbopol^®^-based PPB formulation produced pronounced reductions in preformed biofilm biomass. However, because PB and PPB were incorporated at different concentrations (5% and 1% *w*/*w*, respectively), these findings represent the performance of the final formulations at their selected formulation-specific concentrations and should not be interpreted as an equivalent-dose comparison of the intrinsic biological potency of the two pharmabiotic fractions. These findings provide an initial physicochemical and in vitro biological characterization of *L. casei*-derived pharmabiotic gel-serums and support their further investigation as topical formulation platforms. The present study does not establish long-term storage stability or dermocosmetic or dermatological applicability. Further studies should therefore include systematic storage-stability and release studies, detailed compositional characterization of the PB and PPB preparations, cytocompatibility testing using relevant skin cells, irritation and sensitization assessments, reconstructed human skin models, and ex vivo permeation/penetration studies. These studies are needed to establish the long-term performance, skin-relevant safety, and potential topical applicability of the developed formulations.

## 4. Materials and Methods

### 4.1. Materials

*Lacticaseibacillus casei*-derived postbiotic and paraprobiotic fractions used in this study were prepared through controlled fermentation and inactivation procedures performed within the scope of the present project. Medium molecular weight (MMW) chitosan (Sigma-Aldrich, St. Louis, MO, USA) and Carbopol^®^ 940 (Zag Kimya, İstanbul, Türkiye) were employed as natural and synthetic gelling polymers, respectively. Citric acid monohydrate (Merck, Darmstadt, Germany) was used for chitosan solubilization, whereas sorbitol (Sigma-Aldrich, USA), polyethylene glycol 400 (PEG 400; Merck, Germany), and urea (Merck, Germany) were incorporated as humectants and formulation auxiliaries. Polysorbate 20 (Sigma-Aldrich, USA) served as the surfactant, while sodium salicylate (Sigma-Aldrich, USA) and ammonium benzoate (Merck, Germany) were included as preservative components. Triethanolamine (Merck, Germany) was used to neutralize Carbopol^®^ formulations. Ultrapure water obtained from a laboratory purification system was used throughout the study. All chemicals and reagents were of analytical grade and were used without further purification.

### 4.2. Preparation of Postbiotic and Paraprobiotic Fractions

#### 4.2.1. Preparation of Postbiotic Fraction

*Lacticaseibacillus casei* was cultivated in de Man–Rogosa–Sharpe (MRS) broth at 37 ± 1 °C for 48 h. Following incubation, the cultures were centrifuged at 4000 rpm for 20 min to separate bacterial cells from the culture supernatant. The resulting cell-free supernatant, containing extracellular metabolites, was carefully collected and sterilized by filtration through a 0.22-µm membrane filter [40]. The sterile supernatant was frozen at −40 °C for 24 h and subsequently freeze-dried using a laboratory lyophilizer(Labconco Corporation, Kansas City, MO, USA) operated at a freezing temperature of −40 °C, shelf temperature of −60 °C, and chamber pressure of 100 mTorr. The obtained postbiotic powder was stored at −20 °C until further use in formulation development and biological analyses. To improve reproducibility, the preparation procedure was standardized with respect to the initial bacterial culture conditions, processing procedure, and lyophilized fraction used for formulation. The PB fraction was obtained from the cell-free fraction following removal of bacterial cells and was subsequently lyophilized under standardized conditions. The resulting lyophilized preparation was used on a weight basis for formulation and was incorporated at 5% (*w*/*w*).

#### 4.2.2. Preparation of Paraprobiotic Fraction

For paraprobiotic production, *L. casei* cultures were grown under identical conditions in MRS broth at 37 ± 1 °C for 48 h. After incubation, cultures were centrifuged at 4000 rpm for 20 min, and the resulting cell pellets were collected. Pellets were washed twice with sterile phosphate-buffered saline (PBS) to remove residual extracellular components. The washed bacterial suspension was thermally inactivated by incubation at 80 °C for 30 min [41]. Following heat treatment, samples were allowed to cool to room temperature, frozen at −40 °C for 24 h, and lyophilized under the same operating conditions used for postbiotic preparation (freezing temperature: −40 °C, shelf temperature: −60 °C, chamber pressure: 100 mTorr). The freeze-dried paraprobiotic powder was collected under sterile conditions and stored at −20 °C until use. The PPB preparation was similarly produced under standardized processing conditions and lyophilized before incorporation into the formulations. The resulting lyophilized preparation was used on a weight basis and incorporated at 1% (*w*/*w*). Successful microbial inactivation was confirmed by culture-based viability testing on MRS agar and Nutrient Agar, with no colony formation observed following incubation.

### 4.3. Verification of Microbial Inactivation

Successful microbial inactivation was confirmed by viability testing. Freeze-dried postbiotic and paraprobiotic powders were reconstituted in sterile distilled water, and aliquots were spread onto MRS agar and Nutrient Agar plates. The inoculated plates were incubated at 37 °C for 24 h, after which the absence of visible colony formation confirmed complete microbial inactivation.

### 4.4. Preliminary Optimization of Polymer Concentrations

Prior to preparation of the final pharmabiotic gel-serums, preliminary formulation screening was performed to select suitable polymer concentrations. Carbopol^®^ was evaluated at concentrations of 0.10, 0.15, 0.20, 0.25, and 0.30% (*w*/*w*), whereas chitosan was evaluated at concentrations of 0.10, 0.20, 0.30, 0.40, and 0.50% (*w*/*w*). All formulations were prepared under otherwise comparable conditions and were qualitatively evaluated based on macroscopic appearance, homogeneity, gel formation, consistency, ease of handling, and ease of spreading. Formulations exhibiting insufficient structural consistency or excessively high consistency with unfavorable handling and spreading characteristics were excluded. The polymer concentration providing the most suitable balance of structural integrity, homogeneity, and handling/spreading characteristics was selected for subsequent formulation development.

### 4.5. Preparation of Pharmabiotic Gel-Serums

The different incorporation levels of postbiotic (PB) and paraprobiotic (PPB) were selected based on preliminary formulation considerations and the distinct physical characteristics of the two lyophilized preparations. These concentrations were chosen to obtain technically suitable gel-serum formulations rather than to provide equivalent amounts of the two pharmabiotic fractions. Accordingly, PB was incorporated at 5% (*w*/*w*), whereas PPB was incorporated at 1% (*w*/*w*).

#### 4.5.1. Preparation of Carbopol^®^-Based Gel-Serums

Carbopol^®^-based gel-serums were prepared according to the method described by Öztürk et al. (2018) with slight modifications. Carbopol^®^ 940 was gradually dispersed in ultrapure water under continuous magnetic stirring until complete hydration was achieved [42]. Sorbitol, polyethylene glycol 400 (PEG 400), urea, sodium salicylate, ammonium benzoate, and Polysorbate 20 were subsequently incorporated into the hydrated polymer dispersion to obtain a homogeneous gel base. Gelation was achieved by the controlled addition of triethanolamine (TEA), which simultaneously neutralized the polymer and adjusted the formulation to a skin-compatible pH. Finally, either the postbiotic or paraprobiotic powder was incorporated into the blank gel base under gentle stirring until complete and homogeneous distribution was achieved. The prepared formulations were stored under appropriate conditions until physicochemical characterization and biological evaluation.

#### 4.5.2. Preparation of Chitosan-Based Gel-Serums

Chitosan gel-serums were prepared using medium molecular weight chitosan according to Yenilmez et al. (2015) with minor modifications [43]. Citric acid monohydrate was first dissolved in ultrapure water to provide an acidic medium suitable for chitosan dissolution. Chitosan powder was then gradually added under continuous magnetic stirring until a homogeneous polymer solution was obtained. The dispersion was allowed to hydrate completely at room temperature. Following complete hydration, sorbitol, PEG 400, urea, sodium salicylate, ammonium benzoate, and Polysorbate 20 were sequentially incorporated into the formulation. Finally, postbiotic or paraprobiotic powder was dispersed into the gel base under gentle stirring to ensure homogeneous distribution. The pH of each formulation was measured and adjusted when necessary before storage for further analyses.

### 4.6. Physicochemical Characterization of Pharmabiotic Gel-Serums

#### 4.6.1. Rheological Analysis

The rheological properties of blank formulations and pharmabiotic gel-serums containing postbiotic or paraprobiotic fractions were evaluated using a Brookfield rotational rheometer at room temperature. Measurements were performed over a predetermined range of shear rates, and the corresponding shear-stress values were recorded [42]. The flow behavior of each formulation was described using the Herschel–Bulkley rheological model (Equation (1)):

(1) *τ* = *τ*_0_ + *Kγ^n^*

The experimental shear stress–shear rate data were fitted to the Herschel–Bulkley model according to τ = τ_0_ + K*γ*^n^, where τ is the shear stress (Pa), τ_0_ is the yield stress (Pa), K is the consistency index (Pa·s^n^), *γ*^.^ is the shear rate (s^−1^), and n is the flow-behavior index. Model fitting was performed using the rheometer software 3.0 (Brookfield Engineering Laboratories, Inc., Middleboro, MA, USA), and τ_0_, K, and n were determined for each formulation. The goodness of fit was evaluated using both the coefficient of fit (CoF) and coefficient of determination (R^2^). Model fitting was performed according to the procedures described by Ashena et al. (2023) and Mohebbi and Sellier (2022) [44,45,46]. Apparent viscosity values at selected shear rates were calculated from the fitted Herschel–Bulkley parameters according to η_app_ = τ/*γ*^.^, where η_app_ is the apparent viscosity, τ is the shear stress, and *γ*^.^ is the shear rate. Apparent viscosity values were calculated at representative shear rates of 10 and 100 s^−1^.

#### 4.6.2. pH Determination

The pH values of Carbopol^®^- and chitosan-based gel-serums were determined at 25 ± 2 °C using a calibrated digital benchtop pH meter (SevenCompact™ S220, Mettler Toledo, Greifensee, Switzerland). The electrode was immersed directly into each formulation, and measurements were recorded after stabilization of the pH value. All analyses were performed in triplicate, and the results were expressed as mean ± standard deviation (SD) [47].

#### 4.6.3. Spreadability Analysis

The spreadability of the developed gel-serums was determined using a modified glass-slide method adapted from Syakri et al. (2021) [48]. Owing to the limited amount of available formulation, the conventional glass plate system was miniaturized using a standard microscope slide and a square coverslip. Approximately 7 mg of each formulation was placed at the center of the microscope slide, after which the coverslip was carefully positioned over the sample. A calibrated microcentrifuge tube adjusted to a total weight of 1.8 g with distilled water was placed on the coverslip for 1 min to provide a constant compressive force. After removal of the weight, the diameters of the spread formulation were measured along two perpendicular axes (D_1_ and D_2_) using a digital caliper. The mean spreading diameter and corresponding spreading area were subsequently calculated for each formulation. Measurements were performed in triplicate.

#### 4.6.4. Macroscopic Evaluation

Freshly prepared gel-serums were visually inspected at room temperature to evaluate their macroscopic characteristics. Formulations were examined for color, appearance, transparency, homogeneity, consistency, phase separation, precipitation, agglomeration, and the presence of visible foreign particles. Observations were recorded immediately after formulation preparation and comparatively evaluated to assess the physical quality of each gel-serum [49].

### 4.7. Biological Evaluation of Pharmabiotic Gel-Serums

#### 4.7.1. Antimicrobial Activity

The antimicrobial activity of postbiotic- and paraprobiotic-loaded gel-serums was evaluated using the agar well diffusion and broth microdilution methods. *Bacillus subtilis* NRRL B-4378, *Staphylococcus aureus* ATCC 29213, *Pseudomonas aeruginosa* ATCC 27853, *Candida albicans* ATCC 90028, and *Candida krusei* ATCC 6258 were used as test microorganisms. To distinguish the contribution of the polymeric vehicle from that of the incorporated pharmabiotic fractions, blank Carbopol^®^ (CA-C-Blank) and blank chitosan (CA-CS-Blank) formulations, free postbiotic (PB) and paraprobiotic (PPB) fractions, and the corresponding complete formulations (CA-C-PB, CA-C-PPB, CA-CS-PB, and CA-CS-PPB) were included as comparative controls, where applicable.

##### Agar Well-Diffusion Assay

Bacterial strains were cultured on Mueller–Hinton agar (MHA), whereas yeast strains were grown on Sabouraud Dextrose Agar (SDA). Fresh microbial suspensions were adjusted to 0.5 McFarland turbidity (approximately 1.5 × 10^8^ CFU/mL) and uniformly spread onto the corresponding agar plates using sterile cotton swabs. Sterile wells (6 mm) were punched into the agar surface, and 100 μL of each formulation was aseptically dispensed into the wells [50,51]. Chloramphenicol (30 μg) and fluconazole (25 μg) served as positive controls for bacteria and yeasts, respectively. Blank Carbopol^®^ (CA-C-Blank) and chitosan (CA-CS-Blank) formulations, containing the corresponding formulation components without PB or PPB, were included as vehicle controls to assess the antimicrobial contribution of the polymeric matrices and formulation excipients. Following incubation at 37 °C for 24 h for bacteria and 30 °C for 48 h for yeasts, inhibition zone diameters were measured using a digital caliper. All experiments were carried out independently in triplicate, and the results were expressed as mean ± SD. The antimicrobial activity of the free PB and PPB fractions, blank polymeric formulations, and complete pharmabiotic gel-serums was evaluated under the same experimental conditions to allow direct comparison among the groups. Inhibition-zone diameters obtained for the complete formulations were compared directly with those of the corresponding blank formulations and free PB or PPB fractions. Blank-associated inhibition zones were considered independently and were not mathematically subtracted from those of the complete formulations, since inhibition-zone diameter may be influenced by both antimicrobial activity and the diffusion characteristics of formulation components within the agar matrix.

##### Minimum Inhibitory Concentration (MIC)

Minimum inhibitory concentrations (MICs) of the postbiotic and paraprobiotic fractions and their corresponding gel-serums were determined using the broth microdilution method according to the Clinical and Laboratory Standards Institute (CLSI M07 and CLSI M27) guidelines. All tested preparations were evaluated using the same concentration range. Serial two-fold dilutions were prepared from an initial concentration of 10,000 μg/mL in sterile 96-well microplates containing Mueller–Hinton broth for bacterial strains and RPMI-1640 medium for *Candida* species. Microbial inocula were adjusted to final concentrations of 5 × 10^5^ CFU/mL for bacteria and 0.5–2.5 × 10^3^ CFU/mL for yeasts. The plates were incubated at 37 °C for 24 h for bacteria and at 35 °C for 48 h for yeasts. Following incubation, 0.01% resazurin solution was added to each well, and color changes were visually evaluated. The lowest tested concentration showing complete inhibition of microbial growth was recorded as the MIC [50,52]. All assays were performed independently in triplicate. MIC values were determined for the free PB and PPB fractions, blank Carbopol^®^ (CA-C-Blank) and chitosan (CA-CS-Blank) formulations, and the corresponding complete PB- and PPB-containing formulations. The blank formulations were evaluated using the same serial dilution range and experimental conditions as the corresponding complete formulations and served as vehicle controls to assess the potential contribution of the polymeric matrices and formulation excipients to microbial growth inhibition. MIC values of the complete formulations were directly compared with those of the corresponding blank formulations and free PB or PPB fractions. Blank MIC values were considered independently and were not mathematically subtracted from those of the complete formulations. In addition, microorganism-free sample control wells containing the corresponding formulations were included to account for potential interference of formulation color, turbidity, or precipitation with the resazurin-based endpoint assessment.

#### 4.7.2. Antibiofilm Activity

The antibiofilm activity of the developed pharmabiotic gel-serums was evaluated using the crystal-violet microplate assay [52]. *Staphylococcus aureus* ATCC 29213 and *Pseudomonas aeruginosa* ATCC 27853 were selected as representative biofilm-forming microorganisms. Bacterial suspensions were prepared in Tryptic Soy Broth (TSB) supplemented with 1% (*w*/*v*) glucose and adjusted to the 0.5 McFarland turbidity standard. Aliquots (200 μL) were transferred into sterile flat-bottom 96-well microplates and incubated at 37 °C for 24 h to allow biofilm formation. Following incubation, planktonic cells were carefully removed, and the wells were gently washed three times with sterile phosphate-buffered saline (PBS). Subsequently, the postbiotic-, paraprobiotic-, and pharmabiotic gel-serum formulations were added to the established biofilms, followed by incubation at 37 °C for an additional 24 h. After treatment, the wells were washed three times with PBS to remove non-adherent cells. The remaining biofilms were fixed with 99% methanol, stained with 0.1% crystal-violet solution for 15 min, and washed thoroughly with distilled water to remove excess stain. The bound crystal violet was then solubilized with ethanol, and absorbance was measured at 570 nm using a microplate reader. The effects of free PB and PPB fractions, blank Carbopol^®^ (CA-C-Blank) and chitosan (CA-CS-Blank) formulations, and the corresponding complete PB- and PPB-containing formulations were evaluated against preformed biofilms under identical experimental conditions. The blank formulations, containing the corresponding formulation components without PB or PPB, were included as vehicle controls to determine the contribution of the polymeric matrices and formulation excipients to changes in preformed biofilm biomass. The percentage reductions obtained for the blank formulations were evaluated independently and directly compared with those of the corresponding complete formulations. Blank-associated reductions were not mathematically subtracted from those of the complete formulations, since the effects of the polymeric matrix, excipients, and incorporated pharmabiotic fractions may not be strictly additive. The reduction in preformed biofilm biomass was calculated relative to the untreated biofilm control according to the following equation (Equation (2)):

(2) $$\mathrm{Biofilm}\;\mathrm{Inhibition}\;(\%)=\frac{OD_{control}\;-\;OD_{sample}}{OD_{control}}\times 100$$

where *OD_control_* represents the absorbance of untreated preformed biofilms and *ODs_ample_* represents the absorbance of preformed biofilms following treatment with the corresponding free fraction, blank formulation, or complete formulation. All experiments were performed independently in triplicate, and the results were expressed as mean ± standard deviation (SD).

#### 4.7.3. DPPH Radical-Scavenging Activity

The antioxidant capacity of postbiotic, paraprobiotic, and pharmabiotic gel-serum formulations was determined using the 2,2-diphenyl-1-picrylhydrazyl (DPPH) free-radical-scavenging assay. The samples were prepared, and 100 μL of each solution was mixed with an equal volume (100 μL) of freshly prepared 0.1 mM DPPH methanolic solution in a 96-well microplate. The reaction mixtures were incubated in the dark at room temperature for 30 min. Following incubation, absorbance values were measured at 517 nm using a microplate reader [53]. Free PB and PPB fractions, blank polymeric formulations, and complete pharmabiotic gel-serums were evaluated under the same conditions to assess the respective contributions of the microbial-derived fractions and formulation matrices to DPPH radical-scavenging activity. The blank Carbopol^®^ (CA-C-Blank) and chitosan (CA-CS-Blank) formulations were included as vehicle controls and evaluated under the same experimental conditions as the corresponding complete formulations. The DPPH radical-scavenging activities of the blank formulations were evaluated independently and directly compared with those of the corresponding PB- and PPB-containing formulations. Blank-associated activity was not mathematically subtracted from that of the complete formulations. Radical-scavenging activity was calculated using the following equation (Equation (3)):

(3) $$\mathrm{DPPH}\;\mathrm{Inhibition}\;\left(\%\right)=\frac{A_{control}\;-\;A_{sample}}{A_{control}}\times 100$$

where *A_control_* is the absorbance of the control solution and *A_sample_* is the absorbance of the tested sample.

### 4.8. Statistical Analysis

Each formulation was independently prepared in three separate batches (n = 3). Where applicable, analytical and biological measurements were performed in technical replicates within each independently prepared batch, with the independent formulation batch considered as the experimental unit. Results are expressed as mean ± standard deviation (SD). Blank Carbopol^®^ (CA-C-Blank) and chitosan (CA-CS-Blank) formulations were treated as independent vehicle-control groups in the statistical analyses and were compared with the corresponding PB- and PPB-containing formulations, as appropriate. Statistical analyses were performed using GraphPad Prism version 8 software (GraphPad Software Inc., San Diego, CA, USA), and the normality of data distribution was assessed prior to statistical comparisons. For outcomes involving the factorial formulation structure, two-way analysis of variance (ANOVA) was used to evaluate the main effects of polymer type and pharmabiotic fraction, as well as their interaction. Tukey’s multiple comparison test was applied for post hoc comparisons where appropriate. For analyses not involving the factorial formulation structure, one-way ANOVA followed by Tukey’s multiple comparison test was used as appropriate. Student’s *t*-test was used for comparisons between two independent groups when applicable. Differences were considered statistically significant at *p* < 0.05.

## Figures and Tables

**Table 1:** Preliminary screening of Carbopol^®^ and chitosan concentrations for selection of the polymeric gel-serum bases.

Polymer	Concentration (% *w*/*w*)	Preliminary Observation	Decision
Carbopol^®^	0.10	Low structural consistency	Not selected
Carbopol^®^	0.15	Low structural consistency	Not selected
Carbopol^®^	0.20	Improved but insufficient consistency	Not selected
Carbopol^®^	0.25	Homogeneous; suitable consistency and spreading characteristics	Selected
Carbopol^®^	0.30	Higher consistency; less favorable handling/spreading	Not selected
Chitosan	0.10	Insufficient consistency	Not selected
Chitosan	0.20	Improved but insufficient consistency	Not selected
Chitosan	0.30	Homogeneous; suitable consistency and spreading characteristics	Selected
Chitosan	0.40	High consistency; reduced ease of spreading	Not selected
Chitosan	0.50	Excessive consistency; unfavorable handling/spreading	Not selected

**Table 2:** Composition of Carbopol^®^-based pharmabiotic gel-serum formulations (% *w*/*w*).

Ingredient	CA-C-Blank	CA-C-PB	CA-C-PPB
Carbopol^®^ 940	0.25	0.25	0.25
Sorbitol	3.00	3.00	3.00
PEG 400	2.00	2.00	2.00
Urea	1.00	1.00	1.00
Sodium salicylate	0.50	0.50	0.50
Ammonium benzoate	0.30	0.30	0.30
Polysorbate 20	0.10	0.10	0.10
Triethanolamine	q.s.	q.s.	q.s.
Postbiotic	–	5.00	–
Paraprobiotic	–	–	1.00
Purified water	q.s. to 100	q.s. to 100	q.s. to 100

q.s.: quantity sufficient.

**Table 3:** Composition of chitosan-based pharmabiotic gel-serum formulations (% *w*/*w*).

Ingredient	CA-CS-Blank	CA-CS-PB	CA-CS-PPB
Medium molecular weight chitosan	0.30	0.30	0.30
Citric acid monohydrate	0.30	0.30	0.30
Sorbitol	3.00	3.00	3.00
PEG 400	2.00	2.00	2.00
Urea	1.00	1.00	1.00
Sodium salicylate	0.50	0.50	0.50
Ammonium benzoate	0.30	0.30	0.30
Polysorbate 20	0.10	0.10	0.10
Postbiotic	–	5.00	–
Paraprobiotic	–	–	1.00
Purified water	q.s. to 100	q.s. to 100	q.s. to 100

**Table 4:** Herschel–Bulkley rheological parameters of the developed pharmabiotic gel-serums.

Formulation	Consistency Index (K)	Flow-Behavior Index (n)	Yield Stress (Pa)	Apparent Viscosity at 10 s^−1^ (Pa·s)	Apparent Viscosity at 100 s^−1^ (Pa·s)	R^2^	CoF (%)
CA-C-Blank	653.8	0.52	−5.35	215.96	71.63	1.000	100.0
CA-C-PB	158.9	0.74	1.22	87.44	48.00	0.999	99.9
CA-C-PPB	136.0	0.78	1.69	82.12	49.40	1.000	100.0
CA-CS-Blank	249.9	0.57	14.00	94.25	34.64	0.994	99.4
CA-CS-PB	362.7	0.56	3.27	132.02	47.85	0.995	99.5
CA-CS-PPB	196.8	0.72	−0.93	103.19	54.19	1.000	100.0

**Table 5:** Summary of the rheological behavior of the developed pharmabiotic gel-serum formulations.

Formulation	Flow Behavior	Rheological Characteristics
CA-C-Blank	Pseudoplastic	Highest consistency index among Carbopol^®^-based formulations
CA-C-PB	Pseudoplastic	Reduced consistency index compared with the blank formulation
CA-C-PPB	Pseudoplastic	Lowest consistency index among Carbopol^®^-based formulations
CA-CS-Blank	Pseudoplastic	Highest yield stress among all formulations
CA-CS-PB	Pseudoplastic	Highest consistency index among chitosan-based formulations
CA-CS-PPB	Pseudoplastic	Lower consistency index than the postbiotic-loaded chitosan formulation

**Table 6:** pH values of the developed Carbopol^®^- and chitosan-based pharmabiotic gel-serum formulations (mean ± SD, n = 3).

Formulation	pH
CA-C-Blank	4.40 ± 0.02
CA-C-PB	4.23 ± 0.05
CA-C-PPB	5.66 ± 0.01
CA-CS-Blank	5.51 ± 0.04
CA-CS-PB	4.22 ± 0.01
CA-CS-PPB	5.64 ± 0.03

**Table 7:** Spreadability parameters of the developed pharmabiotic gel-serum formulations.

Formulation	D_1_ (mm)	D_2_ (mm)	Mean Diameter (mm)	Spreading Area (mm^2^)
CA-C-Blank	18.6	18.1	18.35	264.4
CA-C-PB	19.7	19.3	19.50	298.6
CA-C-PPB	20.4	20.3	20.35	325.3
CA-CS-Blank	16.5	16.2	16.35	209.9
CA-CS-PB	15.4	15.1	15.25	182.7
CA-CS-PPB	17.3	17.1	17.20	232.2

**Table 8:** Macroscopic characteristics of the developed pharmabiotic gel-serum formulations.

Formulation	Color	Appearance	Homogeneity	Phase Separation	Precipitation	Consistency
CA-CS-Blank	Colorless	Slightly opalescent	+	None	None	Serum-like
CA-CS-PB	Brown	Homogeneous	+	None	None	Serum-like
CA-CS-PPB	Cream–white	Slightly opaque	+	None	None	Serum-like
CA-C-Blank	Colorless	Transparent	+	None	None	Serum-like
CA-C-PB	Brown	Homogeneous	+	None	None	Serum-like
CA-C-PPB	Cream–white	Slightly opaque	+	None	None	Serum-like

+, homogeneous.

**Table 9:** Minimum inhibitory concentrations (MICs) of *Lacticaseibacillus casei*-derived postbiotic and postbiotic-loaded pharmabiotic gel-serum formulations (µg/mL).

Sample	*B. subtilis* NRRL B478	*S. aureus* ATCC 29213	*P. aeruginosa* ATCC 27853	*C. albicans* ATCC 90028	*C. krusei* ATCC 6258
Purified postbiotic	2500	5000	2500	5000	5000
CA-C-Blank	>5000	>5000	>10,000	>5000	>5000
CA-CS-Blank	>5000	>5000	>5000	>2500	>2500
CA-C-PB Postbiotic-loaded Carbopol^®^	3125	3125	3125	1156	1156
CA-CS-PB Postbiotic-loaded Chitosan	3125	3125	3125	1156	1156
Erythromycin	0.0019	0.0019	–	–	–
Ciprofloxacin	–	–	0.0005	–	–
Fluconazole	–	–	–	0.0312	0.0312

**Table 10:** Minimum inhibitory concentrations (MICs) of *Lacticaseibacillus casei*-derived paraprobiotic and paraprobiotic-loaded pharmabiotic gel-serum formulations (µg/mL).

Sample	*B. subtilis* NRRL B478	*S. aureus* ATCC 29213	*P. aeruginosa* ATCC 27853	*C. albicans* ATCC 90028	*C. krusei* ATCC 6258
Purified paraprobiotic	10,000	10,000	10,000	5000	5000
CA-C-Blank	>5000	>5000	>10,000	>5000	>5000
CA-CS-Blank	>5000	>5000	>5000	>2500	>2500
CA-C-PPB Paraprobiotic-loaded Carbopol^®^	–	–	3125	3125	3125
CA-CS-PPB Paraprobiotic-loaded Chitosan	6250	3125	3125	3125	3125
Erythromycin	0.0019	0.0019	–	–	–
Ciprofloxacin	–	–	0.0005	–	–
Fluconazole	–	–	–	0.0312	0.0312

## Data Availability

The data supporting the findings of this study are available from the corresponding author upon reasonable request.

## Abbreviations

The following abbreviations are used in this manuscript:

PB	Postbiotic
PPB	Paraprobiotic
CA-C-Blank	Carbopol^®^-based blank gel-serum formulation
CA-C-PB	Carbopol^®^-based postbiotic-loaded gel-serum formulation
CA-C-PPB	Carbopol^®^-based paraprobiotic-loaded gel-serum formulation
CA-CS-Blank	Chitosan-based blank gel-serum formulation
CA-CS-PB	Chitosan-based postbiotic-loaded gel-serum formulation
CA-CS-PPB	Chitosan-based paraprobiotic-loaded gel-serum formulation
MIC	Minimum Inhibitory Concentration
DPPH	2,2-Diphenyl-1-picrylhydrazyl
MHA	Mueller–Hinton Agar
MHB	Mueller–Hinton Broth
SDA	Sabouraud Dextrose Agar
TSB	Tryptic Soy Broth
PBS	Phosphate-Buffered Saline
RPMI	Roswell Park Memorial Institute
OD	Optical Density
CFU	Colony-Forming Unit
CLSI	Clinical and Laboratory Standards Institute
CoF	Coefficient of Fit

## References

[1] Naldi L. (2004). Epidemiology of Psoriasis. Curr. Drug Targets Inflamm. Allergy.

[2] Ryu K.A., Park P.J., Kim S.B., Bin B.H., Jang D.J., Kim S.T. (2020). Topical Delivery of Coenzyme Q10-Loaded Microemulsion for Skin Regeneration. Pharmaceutics.

[3] Costerton J.W., Stewart P.S., Greenberg E.P. (1999). Bacterial Biofilms: A Common Cause of Persistent Infections. Science.

[4] Draelos Z.K. (1995). Cosmetics: An Overview. Curr. Probl. Dermatol..

[5] Üstündağ Okur N., Karadağ A.E., Ipekçi E., Özcan Bülbül E. (2020). Kozmetik Preparatlar ve Kozmetik Preparatlarda Kullanılan Bitkiler. J. Lit. Pharm. Sci..

[6] Koşar Ş., Ekinci M., Öztürk A.A. (2022). Cilt Tipleri ve İhtiyaçlara Göre Dermakozmetik Ürün Önerilmesinde Eczacının Rolü ve Nanokozmetikler: Geleneksel Derleme. J. Lit. Pharm. Sci..

[7] Güven U.M., Arslan S., Çıracı M.B., Kayıran S.D. (2022). *Calendula officinalis* L. Bitkisinin Morfolojik Özellikleri, Ekstre Içeren Topikal Ilaç Formülasyonu Geliştirilmesi ve in Vitro Değerlendirilmesi. Lokman Hekim Derg..

[8] Salminen S., Collado M.C., Endo A., Hill C., Lebeer S., Quigley E.M.M., Sanders M.E., Shamir R., Swann J.R., Szajewska H. (2021). The International Scientific Association of Probiotics and Prebiotics (ISAPP) Consensus Statement on the Definition and Scope of Postbiotics. Nat. Rev. Gastroenterol. Hepatol..

[9] Aguilar-Toalá J.E., Garcia-Varela R., Garcia H.S., Mata-Haro V., González-Córdova A.F., Vallejo-Cordoba B., Hernández-Mendoza A. (2018). Postbiotics: An Evolving Term within the Functional Foods Field. Trends Food Sci. Technol..

[10] Żółkiewicz J., Marzec A., Ruszczyński M., Feleszko W. (2020). Postbiotics—A Step Beyond Pre- and Probiotics. Nutrients.

[11] Vinderola G., Sanders M.E., Salminen S. (2022). The Concept of Postbiotics. Foods.

[12] Taverniti V., Guglielmetti S. (2011). The Immunomodulatory Properties of Probiotic Microorganisms beyond Their Viability (Ghost Probiotics: Proposal of Paraprobiotic Concept). Genes Nutr..

[13] de Almada C.N., Almada C.N., Martinez R.C.R., Sant’Ana A.S. (2016). Paraprobiotics: Evidences on Their Ability to Modify Biological Responses, Inactivation Methods and Perspectives on Their Application in Foods. Trends Food Sci. Technol..

[14] Moradi M., Kousheh S.A., Almasi H., Alizadeh A., Guimarães J.T., Yılmaz N., Lotfi A. (2020). Postbiotics Produced by Lactic Acid Bacteria: The next Frontier in Food Safety. Compr. Rev. Food Sci. Food Saf..

[15] Compare D., Rocco A., Coccoli P., Angrisani D., Sgamato C., Iovine B., Salvatore U., Nardone G. (2017). *Lactobacillus casei* DG and Its Postbiotic Reduce the Inflammatory Mucosal Response: An Ex-Vivo Organ Culture Model of Post-Infectious Irritable Bowel Syndrome. BMC Gastroenterol..

[16] Rad A.H., Maleki L.A., Kafil H.S., Zavoshti H.F., Abbasi A. (2020). Postbiotics as Novel Health-Promoting Ingredients in Functional Foods. Health Promot. Perspect..

[17] Rad A.H., Aghebati-Maleki L., Kafil H.S., Gilani N., Abbasi A., Khani N. (2021). Postbiotics, as Dynamic Biomolecules, and Their Promising Role in Promoting Food Safety. Biointerface Res. Appl. Chem..

[18] Hao J., Heng P.W.S. (2003). Buccal Delivery Systems. Drug Dev. Ind. Pharm..

[19] Abu-Huwaij R., Assaf S., Salem M., Sallam A. (2007). Potential Mucoadhesive Dosage Form of Lidocaine Hydrochloride: II. In Vitro and in Vivo Evaluation. Drug Dev. Ind. Pharm..

[20] Lee K.Y., Mooney D.J. (2012). Alginate: Properties and Biomedical Applications. Prog. Polym. Sci..

[21] Pérez-Marcos B., Gutiérrez C., Gómez-Amoza J., Martínez-Pacheco R., Souto C., Concheiro A. (1991). Usefulness of Certain Varieties of Carbomer in the Formulation of Hydrophilic Furosemide Matrices. Int. J. Pharm..

[22] Majithiya R.J., Ghosh P.K., Umrethia M.L., Murthy R.S.R. (2006). Thermoreversible-Mucoadhesive Gel for Nasal Delivery of Sumatriptan. AAPS PharmSciTech.

[23] Younes I., Rinaudo M., Harding D., Sashiwa H. (2015). Chitin and Chitosan Preparation from Marine Sources. Structure, Properties and Applications. Mar. Drugs.

[24] Kong M., Chen X.G., Xing K., Park H.J. (2010). Antimicrobial Properties of Chitosan and Mode of Action: A State of the Art Review. Int. J. Food Microbiol..

[25] Nithya S., Nimal T.R., Baranwal G., Suresh M.K., Anju C.P., Anil Kumar V., Gopi Mohan C., Jayakumar R., Biswas R. (2018). Preparation, Characterization and Efficacy of Lysostaphin-Chitosan Gel against *Staphylococcus aureus*. Int. J. Biol. Macromol..

[26] Li C., Fang K., He W., Li K., Jiang Y., Li J. (2021). Evaluation of Chitosan-Ferulic Acid Microcapsules for Sustained Drug Delivery: Synthesis, Characterizations, and Release Kinetics in Vitro. J. Mol. Struct..

[27] Wang Y., Li P., Kong L. (2013). Chitosan-Modified PLGA Nanoparticles with Versatile Surface for Improved Drug Delivery. AAPS PharmSciTech.

[28] Lu B., Lv X., Le Y. (2019). Chitosan-Modified PLGA Nanoparticles for Control-Released Drug Delivery. Polymers.

[29] Boroumand H., Badie F., Mazaheri S., Seyedi Z.S., Nahand J.S., Nejati M., Baghi H.B., Abbasi-Kolli M., Badehnoosh B., Ghandali M. (2021). Chitosan-Based Nanoparticles Against Viral Infections. Front. Cell. Infect. Microbiol..

[30] Theodorou I.M., Kapoukranidou D., Theodorou M., Tsetis J.K., Menni A.E., Tzikos G., Bareka S., Shrewsbury A., Stavrou G., Kotzampassi K. (2024). Cosmeceuticals: A Review of Clinical Studies Claiming to Contain Specific, Well-Characterized Strains of Probiotics or Postbiotics. Nutrients.

[31] Rusic D., Ivic M., Slugan A., Leskur D., Modun D., Durdov T., Vukovic D., Bukic J., Bozic J., Seselja Perisin A. (2024). Pilot Study on the Effects of a Cosmetic Serum Containing Niacinamide, Postbiotics and Peptides on Facial Skin in Healthy Participants: A Randomized Controlled Trial. Life.

[32] Machado P., Ribeiro F.N., Giublin F.C.W., Mieres N.G., Tonin F.S., Pontarolo R., Sari M.H.M., Lazo R.E.L., Ferreira L.M. (2025). Next-Generation Wound Care: A Scoping Review on Probiotic, Prebiotic, Synbiotic, and Postbiotic Cutaneous Formulations. Pharmaceuticals.

[33] Nataraj B.H., Ali S.A., Behare P.V., Yadav H. (2020). Postbiotics-Parabiotics: The New Horizons in Microbial Biotherapy and Functional Foods. Microb. Cell Fact..

[34] Briganti S., Picardo M. (2003). Antioxidant Activity, Lipid Peroxidation and Skin Diseases. What’s New. J. Eur. Acad. Dermatol. Venereol..

[35] Lambers H., Piessens S., Bloem A., Pronk H., Finkel P. (2006). Natural Skin Surface PH Is on Average below 5, Which Is Beneficial for Its Resident Flora. Int. J. Cosmet. Sci..

[36] Proksch E. (2018). PH in Nature, Humans and Skin. J. Dermatol..

[37] Xia W., Liu P., Zhang J., Chen J. (2011). Biological Activities of Chitosan and Chitooligosaccharides. Food Hydrocoll..

[38] Oelschlaeger T.A. (2010). Mechanisms of Probiotic Actions—A Review. Int. J. Med. Microbiol..

[39] Dréno B., Araviiskaia E., Berardesca E., Gontijo G., Sanchez Viera M., Xiang L.F., Martin R., Bieber T. (2016). Microbiome in Healthy Skin, Update for Dermatologists. J. Eur. Acad. Dermatol. Venereol..

[40] Zhao R., Lu Y., Ran J., Li G., Lei S., Zhu Y., Xu B. (2020). Purification and Characterization of Bacteriocin Produced by *Lactobacillus rhamnosus* Zrx01. Food Biosci..

[41] Seong G., Lee S., Min Y.W., Jang Y.S., Kim H.S., Kim E.J., Park S.Y., Kim C.H., Chang D.K. (2021). Effect of Heat-Killed *Lactobacillus casei* DKGF7 on a Rat Model of Irritable Bowel Syndrome. Nutrients.

[42] Öztürk A.A., Güven U.M., Yenilmez E. (2018). Flurbiprofen Loaded Gel Based Topical Delivery System: Formulation and in Vitro Characterization with New Developed UPLC Method. Acta Pharm. Sci..

[43] Yenilmez E., Başaran E., Arslan R., Berkman M.S., Güven U.M., Bayçu C., Yazan Y. (2015). Chitosan Gel Formulations Containing Egg Yolk Oil and Epidermal Growth Factor for Dermal Burn Treatment. Pharmazie.

[44] Ashena R., Badrouchi F., Elmgerbi A., Mishani S., Sotoudeh F., Nekoeian S. (2022). Stepwise Mathematical Derivation of the Herschel–Bulkley Laminar Fluid Flow Equations—In Pipes. J. Pet. Explor. Prod. Technol..

[45] Mohebbi F., Sellier M. (2022). Identification of Rheological Parameters of Herschel–Bulkley Fluids from Free Surface Data. Int. J. Thermofluids.

[46] Soyer P., Öztürk A.A. (2026). Development and Functional Evaluation of a Pharmabiotic Food Supplement Containing Microencapsulated *Lactiplantibacillus pentosus* for Oral Microbiome Health. Food Sci. Nutr..

[47] Miastkowska M., Kulawik-Pióro A., Szczurek M. (2020). Nanoemulsion Gel Formulation Optimization for Burn Wounds: Analysis of Rheological and Sensory Properties. Processes.

[48] Syakri S., Ismail I., Amal N.M., Masjidi N.A., Tahir K.A. (2021). Characterization and Anti-Aging Tests of Peel-Off Gel Masks Made from Ethanolic Extract of Yarrow (*Achillea millefolium*). Open Access Maced. J. Med. Sci..

[49] Dantas M.G.B., Reis S.A.G.B., Damasceno C.M.D., Rolim L.A., Rolim-Neto P.J., Carvalho F.O., Quintans-Junior L.J., Da Silva Almeida J.R.G. (2016). Development and Evaluation of Stability of a Gel Formulation Containing the Monoterpene Borneol. Sci. World J..

[50] Karakaya A., Bağcı E.R., Soyer P., Daoud N.E.H., Erçetin T., Acar Çevik U. (2026). Synthesis, In Vitro Antimicrobial, and Antioxidant Activities of Novel Thiazole Derivatives. Chem. Biodivers..

[51] Solak B., Soyer P., Kaya-Tilki E., Öztürk A.A. (2025). Evaluation of Antimicrobial Properties and Antioxidant Activity of Rosmarinic Acid Loaded Resomer RG 502 H Based Nanoparticles against Tert-Butyl Hydroperoxide-Induced Oxidative Stress: A Detailed Formulation, Characterization and Efficacy Determination Study. J. Drug Deliv. Sci. Technol..

[52] Öğüt K., Özek G., Soyer P., Özek T. (2025). Fatty acid composition and biological activities of *Vitis vinifera* L. (antep karasi) seed oil. J. Fac. Pharm. Ank. Univ..

[53] Hoşer H.C., Soyer P., Tilki E.K., Arısoy S., Alper Öztürk A. (2026). CQA and DoE Guided Development of Brimonidine Tartrate Loaded Polymeric Nanoparticles for Sustained Ocular Delivery and in Vitro Neuroprotective Evaluation in Glaucoma. J. Drug Deliv. Sci. Technol..

